# Mechanical and Ionic Characterization for Organic Semiconductor‐Incorporated Perovskites for Stable 2D/3D Heterostructure Perovskite Solar Cells

**DOI:** 10.1002/smll.202406928

**Published:** 2024-10-07

**Authors:** Jiaonan Sun, Saivineeth Penukula, Muzhi Li, Mona Rasa Hosseinzade, Yuanhao Tang, Letian Dou, Nicholas Rolston

**Affiliations:** ^1^ Davidson School of Chemical Engineering Purdue University West Lafayette IN 47907 USA; ^2^ School of Electrical Computer and Energy Engineering Arizona State University Tempe AZ 85281 USA; ^3^ Birck Nanotechnology Center Purdue University West Lafayette IN 47907 USA

**Keywords:** conjugated cations, ionic characterization, mechanical stability, metal halide perovskites, photovoltaics, 2D materials, reliability

## Abstract

Hybrid metal halide perovskite (MHP) materials, while being promising for photovoltaic technology, also encounter challenges related to material stability. Combining 2D MHPs with 3D MHPs offers a viable solution, yet there is a gap in the understanding of the stability among various 2D materials. The mechanical, ionic, and environmental stability of various 2D MHP ligands are reported, and an improvement with the use of a quater‐thiophene‐based organic cation (4TmI) that forms an organic‐semiconductor incorporated MHP structure is demonstrated. It is shown that the best balance of mechanical robustness, environmental stability, ion activation energy, and reduced mobile ion concentration under accelerated aging is achieved with the usage of 4TmI. It is believed that by addressing mechanical and ion‐based degradation modes using this built‐in barrier concept with a material system that also shows improvements in charge extraction and device performance, MHP solar devices can be designed for both reliability and efficiency.

## Graphical abstract

The mechanical, ionic, and environmental stability of various 2D perovskite ligands is reported. It is shown that the best balance of mechanical robustness, environmental stability, ion activation energy, and reduced mobile ion concentration under accelerated aging is achieved with the usage of 4TmI, a quater‐thiophene‐based organic cation that forms an organic‐semiconductor‐incorporated perovskite structure.

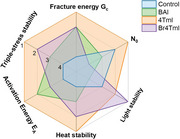

## 1 Introduction

Perovskite materials, especially hybrid metal halide perovskite (MHP), have garnered significant attention because of their enormous potential in the field of solar cells.^[^ ^1^, ^2^ ^]^ However, the further development of perovskite materials has been plagued by their stability challenges.^[^ ^3^, ^4^ ^]^ Because of the “soft” ionic nature of the lattice while being brittle and unable to plastically deform 3D MHP materials are highly susceptible to light, heat, moisture, oxygen, delamination,^[^ ^5^ ^]^ and electric field, etc.^[^ ^6^, ^7^ ^]^ More importantly, because of the relatively weak binding energy between the cations and anions in the lattice, ion migration remains one of the primary degradation pathways.^[^ ^8^, ^9^, ^10^ ^]^

2D MHP materials intercalated with large organic cations have shown improved operational stability.^[^ ^11^, ^12^ ^]^ Therefore, combining 2D and 3D MHP materials together in the form of heterostructures was introduced and this strategy has already contributed to several of the best performing and most stable perovskite solar cell (PSC) devices.^[^ ^12^, ^13^, ^14^, ^15^, ^16^, ^17^ ^]^ BAI, OAI, and PEAI are conventional large organic cations that form Ruddlesden–Popper (RP) phase 2D MHP, and their properties have been widely investigated.^[^ ^18^ ^]^ These materials have also enabled improved bonding and deformability with improved mechanical robustness, an aspect that may contribute to stability improvements.^[^ ^19^ ^]^ Notably, our earlier studies have reported a unique series of quater‐thiophene‐based organic cations, i.e., 4TmI and halogen‐4TmI, which form organic semiconductor‐incorporated perovskite materials (OSiPs).^[^ ^20^, ^21^, ^22^, ^23^ ^]^ Due to the well‐aligned energy level with type‐II alignment for charge extraction, these molecules have enabled PSCs with excellent efficiency and stability.

However, even though 2D MHPs are generally considered more stable than 3D MHPs, the variation of environmental and mechanical stability among different 2D MHP materials is still not well understood. Moreover, there has not been a quantitative understanding of the stability evolution of PSCs incorporating 2D MHPs. In previous work,^[^ ^24^, ^25^ ^]^ we showed that ion migration can be quantified in terms of mobile ion concentration (*N*_o_), a quantity that can give a more complete understanding of the ionic character of MHPs. It would be useful to establish a comprehensive comparison of environmental and mechanical stability among the 2D RP‐phase MHPs with small organic cations and large conjugated cations, and further bridge this 2D material stability and device stability through quantitative characterization.

## 2 Results and Discussion

**Figure 1:**
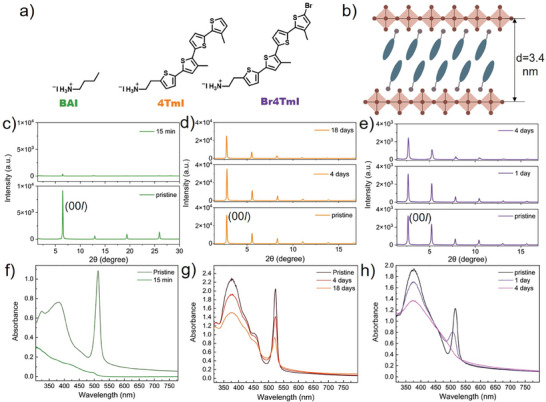
a) Ligand structure of BAI, 4TmI, and Br4TmI b) Schematics of the as‐formed 2D RP phase perovskite. XRD tracking of 2D perovskite thin films for c) (BA)_2_PbI_4_ d) (4Tm)_2_PbI_4_ and e) (Br4Tm)_2_PbI_4_ at 85°C heating under light illumination in air (RH% = 68%). UV–vis spectra tracking of 2D perovskite thin films for f) (BA)_2_PbI_4_, g) (4Tm)_2_PbI_4_, and h) (Br4Tm)_2_PbI_4_ at 85 °C heating under light illumination in air (RH% = 68%).

Here, we compared the stability of 2D RP‐phase MHP materials in which widely used aliphatic BAI, aromatic 4TmI, and Br4TmI (structures in **Figure** **1**a) are incorporated as organic cations, addressing aspects including photo, thermal, atmospheric, ionic, and mechanical stability. The stability of PSC devices based on the above MHPs is evaluated via improvements in ion migration through the quantification of *N*_o_ and calculated activation energy of mobile ions along with thermal and light stability measurements. As a result, the best balance of mechanical robustness, environmental stability, ion activation energy, and reduced *N*_o_ under accelerated aging is achieved with the usage of 4TmI.

2D RP‐phase MHP thin films were fabricated via spin‐coating for initial, film‐level environmental stability characterization. The XRD pattern reveals typical layered structures with calculated d‐spacing as 1.4, 3.2, and 3.4 nm for (BA)_2_PbI_4_, (4Tm)_2_PbI_4_, and (Br4Tm)_2_PbI_4_, respectively (Figure 1c–e). Note that the organic thiophene ligand layers between the PbI_6_ plane are organized together by van der Waal interactions and the distance between the PbI_6_ plane can be expanded as large as 3.4 nm (Figure 1b), more than two times higher compared to the 1.4 nm d‐spacing of BAI 2D perovskites. The UV–vis spectra all reveal excitonic peaks at 513, 523, and 515 nm for (BA)_2_PbI_4_, (4Tm)_2_PbI_4_, and (Br4Tm)_2_PbI_4_, respectively (Figure 1f–h). A rigorous triple‐stress condition combining 85 °C heating, light, and ambient moisture was selected to probe environmental stability. XRD and UV–vis spectra were used to monitor the MHP film degradation under the triple‐stress condition (Figure S1, Supporting Information). For (BA)_2_PbI_4_, accompanied by a rapid film color change from orange to yellow, the diminished XRD and UV–vis peaks clearly show that the whole film degraded within 15 min. The enlarged view also shows the emergence of a new peak at 12.65°, which is attributed to PbI_2_ (Figure S2, Supporting Information). However, for (4Tm)_2_PbI_4_, the stability was much better, as evidenced by XRD patterns that show similar crystallinity even after 18 days of exposure to the harsh triple‐stress conditions. Only from the UV–vis spectra, we can observe a gradual decay of the excitonic peak at 523 nm. For (Br4Tm)_2_PbI_4_, the stability falls in between, revealed from initially a shift of the excitonic peak from 515 to 509 nm, and then a diminished peak after 4 days. We hypothesize that the brominated terminal thiophene in Br4TmI may speed up the photooxidation of iodide in the 2D perovskite lattice and cause degradation. (4Tm)_2_PbI_4_ and (Br4Tm)_2_PbI_4_, with a molecular formula of C_40_H_38_I_4_N_2_PbS_8_ and C_40_H_38_Br_2_I_4_N_2_PbS_8_, respectively, have higher organic content and more bulky structure than conventional (BA)_2_PbI_4_ hybrids, which explains their superior stability performance as 2D RP‐phase perovskite thin films. In summary, 2D MHP with 4TmI demonstrated the best environmental stability, and thiophene‐based 2D MHPs exhibited much better stability compared to conventional BA‐based MHPs.

**Figure 2:**
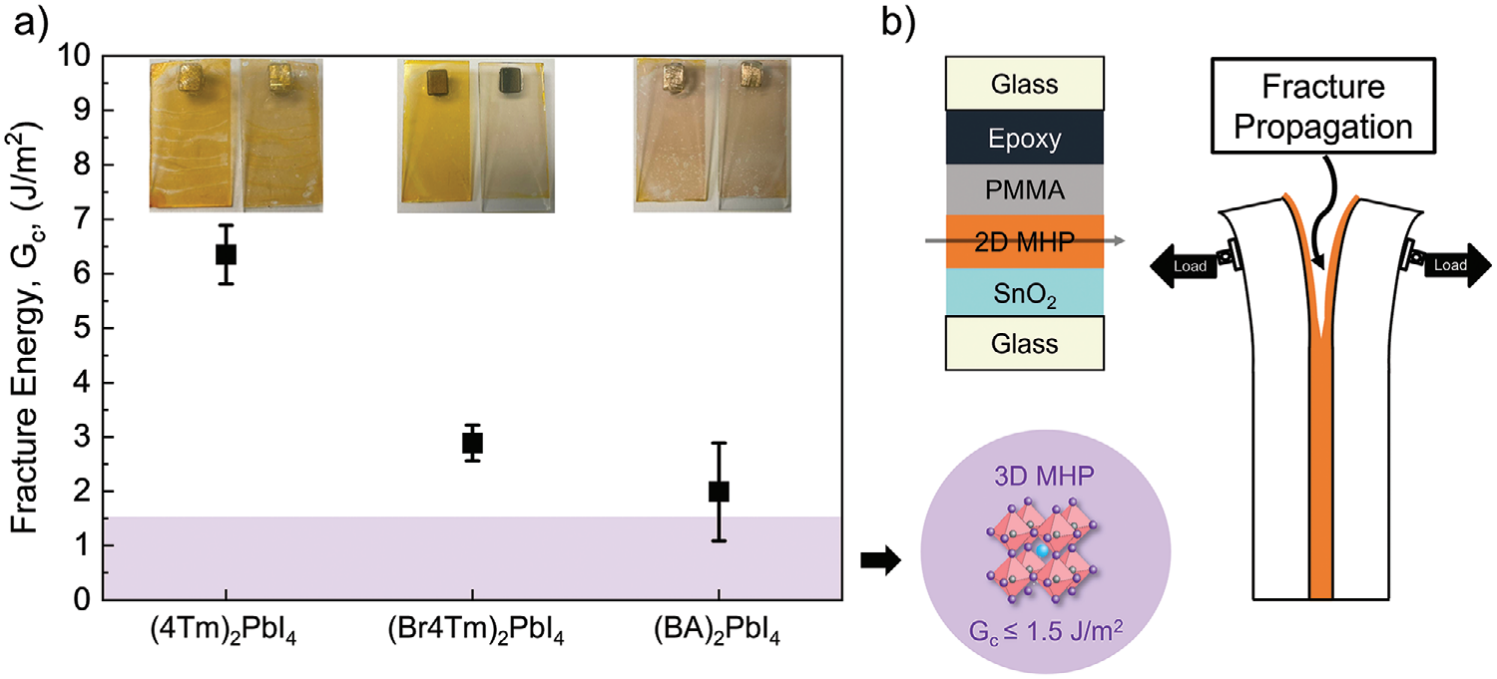
a) Fracture energy of (4Tm)_2_PbI_4_, (Br4Tm)_2_PbI_4_, and (BA)_2_PbI_4_ 2D MHP films, compared to 3D MHPs, insets are representative sample photographs taken after measurements. b) Schematic illustration of samples and fracture propagation for fracture energy measurement using the double cantilever beam (DCB) method.

Mechanical stability of the 2D MHP with ligands BAI, 4TmI, and Br4TmI was quantified through fracture energy (*G*_c_), which has been recognized as a key metric to quantify the reliability of multilayered devices.^[^ ^26^ ^]^ Studies have shown that traditional 3D perovskites, such as MAPbI_3_ and mixed‐cation perovskites (e.g., MA/FA, Cs/FA, and Cs/FA/MA), have low *G*_c_ (≤1.5 J m^−2^) values due to their fragile salt‐like crystal structure.^[^ ^27^ ^]^ With such low *G*_c_ values, PSCs are susceptible to damage from various internal and external stressors, including in‐service stresses caused by mismatches in the thermal expansion coefficients of different layers, as well as from device processing, manufacturing, and installation.^[^ ^28^ ^]^ These factors create a mechanical driving force for damage within the PSCs (*G*), ultimately leading to delamination when *G > G*_c_. Any delamination will then create pathways for accelerated environmental degradation and loss of ohmic contact, resulting in decreased PCE and device failure.^[^ ^19^ ^]^ Therefore, investigating *G*_c_ is crucial for designing mechanically robust PSCs, and achieving robust materials with a high *G*_c_ is essential for extending their operational lifetimes. However, little is known about the mechanical integrity of the emerging 2D perovskites. Our recent work suggested that pure RP‐based perovskites with low n‐values can exceed this low *G*_c_ threshold.^[^ ^19^ ^]^ Here, as shown in **Figure** **2**, we measured the *G*_c_ values of the 4TmI, Br4TmI, and BAI RP‐phase 2D MHPs using a standard fracture configuration known as the double cantilever beam (DCB) test. To conduct the DCB test, we attached an epoxy‐covered top piece of glass to film substrates to create a sandwich‐like structure (Figure 2b) and protected the perovskite from epoxy by using a polymethyl methacrylate coating on top of the film, which was then subjected to uniaxial loading at controlled displacement rates to propagate a crack down the length of the sample. The *G*_c_ results and representative sample photographs after measurements are shown in Figure 2a (raw fracture data and optical images after measurements are shown in Figures S3–S8, Supporting Information). Based on the optical images, perovskite material remained on both sides of the fractured DCB samples, indicating cohesive failure in the measured 2D MHP materials. This enables a direct comparison of *G*_c_ values to determine the mechanical robustness of the 2D MHP materials. The 4Tm‐based 2D MHP has a *G*_c_ of 6.35 ± 0.54 J m^−2^, which is the highest of any unmodified MHP that has been measured to date, and it is significantly higher than the measured Br4Tm‐based (*G*_c_ = 2.9 ± 0.32 J m^−2^) and BA‐based (*G*_c_ = 1.99 ± 0.90 J m^−2^) 2D MHPs. Compared to (BA)_2_PbI_4_, the better cohesion of 2D (4Tm)_2_PbI_4_ and (Br4Tm)_2_PbI_4_ can be attributed to their large cations, likely allowing improved film morphology, plasticity, and capability to deform.^[^ ^29^ ^]^ We also hypothesize that the reduced *G*_c_ of (Br4Tm)_2_PbI_4_ could originate from the smaller grain size and more grain boundaries induced by using Br4TmI as organic cations, which can weaken the layer interactions and initiate more defects to initiate fracture when subjected to tensile stress.^[^ ^28^, ^30^ ^]^ To confirm our hypothesis, we analyzed scanning electron microscope (SEM) images, as shown in Figure S9 (Supporting Information). (BA)_2_PbI_4_ exhibits a rough, radiative needle‐like surface structure, well aligned with its weakest fracture energy. (Br4Tm)_2_PbI_4_ reveals a very small grain size, which makes it difficult to discern grain boundaries from SEM images alone. The presence of round‐shape aggregates can be assigned to extra Br4TmI ligands, indicating a slightly lower propensity to form 2D MHPs. The extra Br4TmI may introduce defects and elevate the initial *N*_o_, as will be shown in the next section. Conversely, for (4Tm)_2_PbI_4_, SEM images reveal a relatively large grain size, ≈7 µm, which explains its ability to achieve the highest *G*_c_. Overall, the same trend was observed in *G*_c_ as in the triple‐stress environmental stability for the materials, where the 4TmI is the most robust followed by the Br4TmI and the BAI.

**Figure 3:**
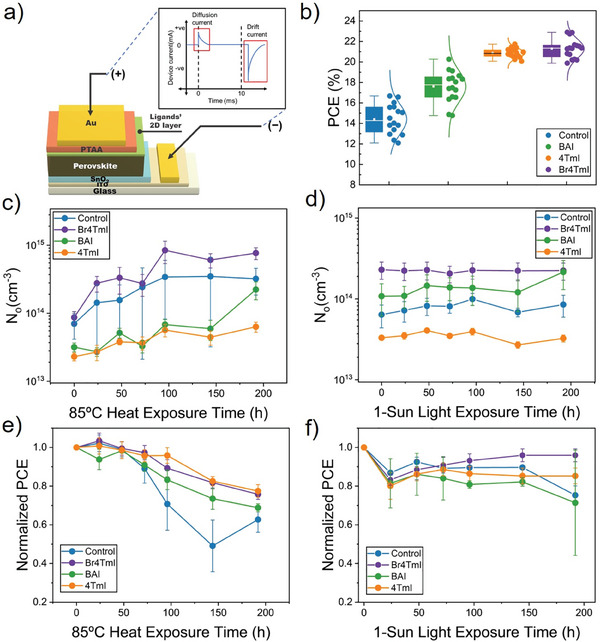
a) Schematic illustration of perovskite solar cell configurations and mobile ion concentration (*N_o_*) measurement. b) Statistics of PCE based on devices without interlayers, with BAI‐2D, with 4TmI‐2D, and with Br4TmI‐2D interlayers. Statistics are from 16 devices. c) *N*_o_ evolution under 85 °C heat exposure. d) *N*_o_ evolution under light exposure. e) Device PCE evolution under 85 °C heat exposure. f) Device PCE evolution under light exposure.

After investigating the stability performance of 2D MHP materials, n‐i‐p PSCs with 2D/3D heterostructures were fabricated to further probe the ionic properties, as well as tracking the evolution of ionic characteristics with accelerated heat (85 °C) and light (continuous 1 sun) exposure. A standard device structure, glass/ITO/SnO_2_/MHP/2D layer/PTAA/Au, is shown in **Figure** **3**a. The absorption and photoluminescence spectra of FA_0.9_MA_0.05_Cs_0.05_PbI_3_ MHP absorber layer fabricated via a two‐step method are included in Figure S10 (Supporting Information). The devices that do not have any 2D MHP interlayer are considered as controls. Compared with control devices with an average power conversion efficiency (PCE) of 14%, the addition of a 2D interlayer clearly enhanced the PCE of solar cells (Figure 3b), mainly due to the enhanced fill factor and open circuit voltage (Figures S11–S16, Supporting Information). Thiophene‐based solar cell devices, 4TmI and Br4TmI, reveal an improved device PCE of over 21% compared to the 18% PCE of BAI‐based solar cells. Especially, the highest efficiency for 4TmI and Br4TmI reached 21.74% and 22.90%, respectively. This is primarily due to a better energy alignment and enhanced charge transfer from the molecular‐engineered HOMO energy level from the conjugation.^[^ ^23^ ^]^

The PSCs without a 2D interlayer (control) and with a 2D interlayer (Br4TmI, BAI, 4TmI) were aged by subjecting them to 1‐sun light intensity and 85 °C separately (both in N_2_ environments), and *N*_o_ measurements were performed on the PSCs periodically along with PCE measurements to observe the variation of *N*_o_ and PCE in the PSCs with aging. The characterization of *N*_o_ follows the procedure described in our previous work.^[^ ^24^, ^25^ ^]^ In brief, as illustrated in Figure 3a, after a constant bias is applied to the devices in the dark for 10 ms, a transient current response comprising first a diffusion response and subsequently a drift (ionic) current occurred at 0 V during the equilibration process. Drift current, representing the response of the ions in the form of current, was integrated over a millisecond scale to yield *N*_o_, under the assumption and observation that electronic current was swept away from the initial bias. Figure 3c depicts the evolution of *N*_o_ in PSCs with exposure to 85 °C for 192 h. The set of PSCs used for heat aging showed a progressively increasing *N*_o_ in the order of 4TmI, BAI, control, and Br4TmI before exposure. The higher initial *N*_o_ of Br4TmI‐based PSCs could originate from the I^−^ provided by extra Br4TmI present on the surface, as discussed previously in the SEM images. Control PSCs and PSCs with the Br4TmI interlayer showed a continuous increase of *N*_o_ throughout the aging period, while PSC with the BAI interlayer showed significant variation throughout the aging period, ultimately leading to an increase in *N*_o_ at 192 h, and PSCs with the 4TmI interlayer showed the smallest increase in *N*_o_. The exposure to heat was highly influential on *N*_o_ evident from the increase in magnitude of *N*_o_ for all PSCs (also plotted in Figure S17a, Supporting Information). This is also validated in the film‐level aging performed in Figure 1c–h. Figure 3d depicts the evolution of *N*_o_ in PSCs with exposure to light for 192 h. The set of PSCs used for light aging showed a progressively increasing *N*_o_ in the order of 4TmI, control, BAI, and Br4TmI before exposure. Minimal changes were observed in *N*_o_ for all PSCs throughout the 192 h, except for a small increase in *N*_o_ for PSCs with BAI 2D interlayer. Overall, the light exposure was not highly influential on *N*_o_ of PSCs over the period aging was performed (also plotted in Figure S17b, Supporting Information). Figure 3e depicts the evolution of the normalized PCE of PSCs with exposure to 85 °C for 192 h. All the PSCs showed a reduction in PCE throughout the exposure period of 192 h with the PSCs with 4TmI interlayer showing the least reduction with a 23% drop in PCE and the control PSCs showing the largest reduction with a 37% drop in PCE. Hence, we conclude that PSC with 4TmI interlayer is the most stable among the PSCs when exposed to heat with the least variation in *N*_o_ and PCE. Figure 3f depicts the evolution of the normalized PCE of PSCs with exposure to light for 192 h. All the PSCs showed an initial drop in PCE after 24 h with a less significant reduction in PCE over an exposure period of 192 h. The PSCs with Br4TmI interlayer showed the least reduction with a 4% drop in PCE followed by the PSCs with 4TmI interlayer with a 15% drop in PCE, and the PSCs with BAI interlayer showed the most reduction with a 29% drop in PCE. We conclude that PSCs with OSiP interlayers, i.e., Br4TmI‐ and 4TmI‐2D interlayers, are the most stable among the PSCs when exposed to light with the least variation in *N*_o_ and PCE. Also, we proposed that BAI‐2D interlayers, with smaller sizes and “soft” aliphatic chains, could easily penetrate into the lattice of MHP absorbers and have interface reconstruction, which could potentially lead to a fast decay of PCE. Since *N*_o_ measurement reflects changes to the MHP absorber layer itself, some of the less direct correlation between *N*_o_ and stability indicates the degradation mechanism could be driven by electrode and/or charge transport layer changes. It is also important to note that the changes in *N*_o_ of the PSCs are one of the many factors that influence the performance of the PSCs.

The activation energy (*E*_A_) of PSCs was determined using in‐situ ionic conductivity (σ) versus temperature measurements following a method we described previously.^[^ ^24^ ^]^ σ of the PSCs is determined by performing electrochemical impedance spectroscopy (EIS) and extracting ionic resistance from the obtained Nyquist plot by equivalent circuit fitting. E_A_ was then determined using an Arrhenius plot between log(σT) and inverse of temperature (1/T) based on the Equation (1), where σ‐ionic conductivity, T‐temperature in kelvin, K‐Boltzmann constant, E_A_‐activation energy. As shown in Figure S18 (Supporting Information), the E_A_ of the control PSC was 0.166 eV, the E_A_ of the PSC with a Br4TmI 2D interlayer was 0.181 eV, the E_A_ of the PSC with a BAI 2D interlayer was 0.228 eV, and the E_A_ of the PSC with a 4TmI 2D interlayer was 0.234 eV (with fits for E_A_ calculations and in‐situ ionic measurements versus temperature are shown in Figures S19–S23, Supporting Information). It is evident from these values that the introduction of a ligand interlayer in the device structure is improving the formation energy of additional mobile ions when compared to the other PSC architectures. An increase in E_A_ implies that ion formation is suppressed under heating.

(1) $$\;\log\left(\sigma T\right)=\frac{-E_{A}}{K}*\left(1/T\right)$$

Considering the activation energy comparisons along with the triple stress test, mobile ion evolution, and device stability under light and heat, we conclude that 4TmI is the most effective ligand at inhibiting mobile ion formation and MHP degradation.

## 3 Conclusion

As summarized in Table S1 (Supporting Information), it was found that 4TmI‐2D perovskite has the highest stability under extreme conditions that combine light, heat, air, and moisture. Meanwhile, (4Tm)_2_PbI_4_ 2D perovskite has the highest *G*_c_ among all the 2D perovskite materials, much higher than (BA)_2_PbI_4_ and (Br4Tm)_2_PbI_4,_ indicating improved bonding and/or plastic deformation in the 4TmI. Besides, devices with (4Tm)_2_PbI_4_ 2D/3D heterostructures have the lowest *N*_o_ with the highest activation energy of mobile ions. These results highlight that 2D perovskite materials, despite having similar lattice structures, can have large differences in environmental and mechanical stability. The presence of thiophene‐based large, conjugated cations in 2D perovskite can substantially enhance both the environmental and mechanical stability, as well as help decrease *N*_o_ and alleviate ionic migration in the as‐fabricated solar cells. We believe that the key achievements of this work are an improved understanding of the structure‐property relationships, ranging from the stability of 2D perovskite, interlayer mechanical robustness, and the resulting ionic properties in solar cells. The outcomes of this work show a pathway toward the design of MHPs for thermomechanical reliability in addition to performance through control of the structure of 2D perovskite materials for 2D/3D heterostructures. There do remain challenges toward advancing the promise of stable perovskite solar cells with commercially viable lifetimes, a large part of which relies on the lack of validated reliability metrics that are specific to perovskites. We hope that the quantification of mobile ions and mechanical adhesion will be considered key indicators of durable device design.

## 4 Experimental Section

### Ionic Measurements

All the ionic measurements were performed using PAIOS, an all‐in‐one measurement equipment for photovoltaic devices and LEDs. Variation in temperature for determining *E*_A_ was provided by a temperature control stage and module (T96) from Linkam in integration with PAIOS. *N*_o_ was measured using the transient current method as shown in the previous work.^[^ ^24^, ^25^ ^]^ The ionic charge (*Q*_ion_) of the PSCs was measured by letting them equilibrate at 0.8 V for 10 ms in the dark and then the applied bias (*V*_app_) was removed, and the resulting dark transient current was recorded. The drift (ionic) current was considered from the recorded transient current and is integrated over time to obtain *Q*_ion_ of the PSCs. After *Q*_ion_ is obtained, then *N*_o_ is calculated based on Equation (2), where q‐electronic charge, ɛ_o_‐permittivity of free space, ε_r_‐permittivity of material, V_T_‐thermal voltage, V_bi_‐built‐in‐potential, and V_app_‐applied bias (0.8 V).

(2) $$\;Q_{\mathrm{ion}}=\frac{\sqrt{qN_{o}\varepsilon_{o}\varepsilon_{r}V_{T}}}{8}\;*\left[\sqrt{1+16*\left(\frac{V_{bi}}{V_{T}}\right)}-\sqrt{1+16*\frac{V_{bi}-V_{app}}{V_{T}}}\right]$$

*E*_A_ was determined by measuring ionic conductivity (σ) over a temperature range and measuring the slope of the Arrhenius plot of log (σT) versus the inverse of temperature. σ of the PSCs was determined by performing electrochemical impedance spectroscopy (EIS) on the PSCs and extracting the ionic resistance from the obtained Nyquist plot by equivalent circuit fitting and using Equation (3), where σ‐ionic conductivity, t‐thickness of perovskite, R_i_‐ionic resistance, and A‐area of the electrode.

(3) $$\sigma =\frac{t}{R_{i}*A}\;$$

*E*_A_ of the PSCs was then determined by performing EIS over a temperature range of 300–340K using the temperature control module. Measured σ was plotted in log form versus the inverse of temperature and a linear fit was performed on the plot to extract the slope of the plot, which was used to calculate *E*_A_ based on Equation (1). An LED solar simulator (Newport) was used for aging the PSCs at 1.0 sun AM 1.5G in N_2_ and a hot plate was used to age PSC at 85 °C in an N_2_ glovebox for 192 h with ex‐situ measurements of *N*_o_ using PAIOS. The light was incident on the PSCs through the glass substrate to simulate operational conditions.

### 2D MHP Films Preparation

Organic ligands (0.2 m, 10.6 mg for 4TmI) and PbI_2_ (0.1 m, 4.6 mg) were dissolved in 100 µL DMF/DMSO 4/1 mixed solvents. The mixture is fully dissolved after heating at 70 °C for 2 h. A 1.25 × 1 cm glass substrates were treated with UVO for 15 min before spin coating. Then, 8 µL of the mixed solution was applied onto a glass substrate. Spin‐coating was performed at a speed of 2000 rpm for 30 s, followed by thermal annealing at 150 °C for 10 min (For BAI, thermal annealing is performed at 100 °C). For fracture energy measurement, the glass substrate used was 3 cm × 3 cm. Before coating the 2D MHP, a SnO_2_ layer was coated. For SnO_2_ layer coating, SnO_2_ solution was diluted seven times by mixing 350 µL SnO_2_ aqueous solution (15% in H_2_O), 1050 µL of D.I. H_2_O, and 1050 µL of isopropanol. Then, 100 µL of the diluted SnO_2_ solution was applied onto the large glass substrate, spin‐coated at a speed of 3000 rpm, followed by thermal annealing at 150 °C for 30 min. After SnO_2_ coating, the organic ligand and PbI_2_ mixture were spin‐coated on top, following the same spin‐coating method as the small substrates. To ensure full coverage, 100 µL of the ligand‐PbI_2_ mixed solution was used.

### Fracture Energy Test

*G*_c_ was measured with a standard fracture specimen configuration called a double cantilever beam (DCB). The DCB samples adopted the following structure: glass/SnO_2_/2D MHP/polymethyl methacrylate (PMMA)/epoxy/glass. The dimension of the glass substrate is 30 mm length×15 mm width ×1 mm thickness. PMMA (*M*_W_: ≈3 50 000 g mol^−1^) was dissolved in chlorobenzene (CB) and vortexed to form a PMMA solution (10 wt% in CB). The PMMA layer was deposited to protect the 2D MHP layer from epoxy by spin‐coating the PMMA solution at 3000 rpm for 60 s. Then the as‐prepared samples were left to cure in an N_2_‐filled drybox for 6 h. To create a DCB sample, a layer of thin epoxy (Epo‐Tek 301) was applied to a cover glass superstrate with the identical dimensions as the substrate glass for the device/stack and then bonded to the device/stack to create a sandwich‐like structure with the device layers bonded between glass at room temperature. After 24 h of curing the epoxy in the same dry box, the edges of DCB samples were cleaned to remove the excessive epoxy. Before the fracture energy test was conducted, a pre‐crack was introduced to the DCB samples along the width in order to initiate the crack by inserting the tip of a razor blade in between the two glass substrates of DCB samples. Stainless steel tabs were glued to both sides of the DCB samples for mounting them to a delamination testing system (DTS, USA). In the measurement, the cracked DCB samples were loaded in tension at a constant displacement rate (1 µm s^−1^). When a unit of well‐defined mode I fracture occurred cohesively in the 2D MHP layer, the DCB samples were unloaded and loaded again until a complete separation of the two glass substrates that formed the sandwich‐structured DCB samples was observed. The load (*P*) – displacement (Δ) curves were continuously recorded and used to extract the fracture energy (*G*_c_), which was then calculated and averaged to obtain multiple data points per sample in the following Equation (4):

(4) $$\;G_{c}=\frac{12P_{c}^{2}a^{2}}{B^{2}E^{\prime }h^{3}}\;{\left(1+0.64\frac{h}{a}\right)}^{2}$$

where *P*_c_ is the critical load that deviates from the linear part in the load‐displacement plot during the loading cycle; *a* is the crack length; *B* and *h* are the widths and half height of the sample, respectively; and *E*′ (69 GPa) is the plane‐strain elastic modulus of the glass substrate and superstrate. It is noted that one of the key benefits of this method is that no elastic properties (or thicknesses) of the thin films are needed, which greatly simplifies the analysis. Additionally, the process is identical regardless of the number/thickness of the films assuming they remain much thinner than the substrate thickness of 1 mm, which is always the case for PSCs.

The crack length was estimated by a compliance method:

(5) $$a={\left(\frac{d\Delta }{dP}\cdot \frac{BE^{\prime }h^{3}}{8}\right)}^{\frac{1}{3}}\;-0.64h$$

The *G*_c_ tests were performed under a laboratory air environment.

### Film Characterization

UV–vis spectroscopy was performed on an Agilent Cary‐5000 spectrometer. X‐ray diffraction (XRD) measurements were conducted on a Rigaku Smart Lab using Cu Kα source. The SEM sample substrate is glass fully covered with ITO, then coated with SnO_2_ (using the same method as previously discussed) before applying the 2D MHP coating. The SEM images were captured using a Hitachi S‐4800 SEM operating at a 10.0 kV acceleration voltage with a secondary electron detector.

### 2D/3D Heterostructure PSCs Fabrication

The glass/ITO substrates were cleaned by 15–20 min of sonication in soap water, D.I. water, acetone, isopropanol, acetone (2nd time), and isopropanol (2nd time) sequentially. Before use, the clean substrates were treated with a UVO reactor for 30 min. SnO_2_ was coated on top of the ITO substrate as the first layer. For SnO_2_ layer coating, SnO_2_ solution was diluted seven times by mixing 350 µL SnO_2_ aqueous solution (Alfa Aesar, 15% in H_2_O), 1050 µL of D.I. H_2_O, and 1050 µL of isopropanol. Then, 30 µL of the diluted SnO_2_ solution was applied, spin‐coated at a speed of 3000 rpm, followed by thermal annealing at 150 °C for 30 min. After cooling and 10 min of UVO treatment, a 10 mm KOH solution was applied on top of the SnO_2_ layer via spin coating (3k rpm, 30s) and annealed for 30 min for passivation. For 2‐step perovskite coating with a composition of FA_0.9_MA_0.05_Cs_0.05_PbI_3_, a PbI_2_ solution was prepared by dissolving 691.5 mg PbI_2_ (1.5 m) and 19.5 mg CsI (0.075 m, 5%) in 1 mL DMSO/DMF with 1–9 volume ratio at 70  °C. Cation solution was prepared by dissolving 180 mg FAI (0.52 m), 21.6 mg MACl (0.16 m), and 10 mg MAI (0.03 m) in 2 mL IPA at room temperature. Following 10 min of UVO treatment on the KOH passivated SnO_2_ surface, 35 µL PbI_2_ solution was first spin‐coated onto the substrate and annealed at 70 °C for 1 min (static spin). Then, 100 µL of the cation solution was dispensed onto the PbI_2_‐coated substrates with static spin at 1800 rpm for 30 s. The perovskite films were transferred out of the glove box and annealed at 150 °C in ambient air for 17 min, the environmental humidity was between 40% and 60%. For ligand passivation, all ligand solutions were prepared at a concentration of 0.5 mg mL^−1^, dissolved in a mixed solvent of IPA/CB with a ratio of 1:9. The ligand is dynamically spin‐coated at 4000 rpm for 30 s, and then annealed at 100 °C for 2 min. A PTAA solution was prepared by making 40 mg mL^−1^ solution in chlorobenzene, doped overnight with 11.1 wt% of 4‐isopropyl‐4′‐methyldiphenyliodonium tetrakis(pentafluorophenyl)borate (TPFB) in chlorobenzene at 45 °C. TPFB for doping was prepared at room temperature in a concentration of 100 mg mL^−1^. For spin‐coating, 32 µL of the doped PTAA solution was dynamically coated at 3000 rpm for 30 s, followed by annealing at 80 °C for 5 min. Lastly, 90 nm of gold was thermally evaporated as contact electrodes using a customized shadow mask.

### Device Characterization

*J–V* scans were conducted under calibrated 1.0 sun intensity, with AM 1.5G irradiation based on xenon‐lamp solar simulator (Enlitech SS‐F5‐3A) in the glove box. The light intensity (100 mW cm^−2^) was calibrated each time via a standard Si reference cell certified by NREL. The active area of each device was measured using an Olympus microscope. The reverse scan ranged from 1.2 to −0.1 V, while the forward scan ranged from −0.1 to 1.2 V, with an average scan rate of ≈0.17 V s^−1^. The voltage step is 40 mV from −0.1 to 0.8 V and 10 mV from 0.8 to 1.2 V.

### Statistical Analysis

Preprocessing of the data – normalization was performed on the PCE values of the samples after exposure to heat and light in Figure 3e,f, respectively, by using the ratio PCE_normalized_ = PCE(t)/PCE(t = 0). Data presentation – All the PCE values and G_c_ values in the document are presented as mean ± SD, and the N_o_ values are presented as mean ± SE. Sample size – G_c_ measurements were performed on six samples for each type of 2D MHP film but only the measurements that had valid loading‐unloading curves were included in the calculation of G_c_ of the respective 2D MHP film. So G_c_ for 4TmI films has measurements from four samples, G_c_ for Br4TmI films has measurements from four samples, and G_c_ for BAI films has measurements from three samples. Each device has five pixels on it, *N*_o_ measurements were performed on two such devices for the *N*_o_ versus heat, and *N*_o_ versus light analysis for all the PSCs. That is ten pixels were measured each time for their *N*_o_ and then the mean ± SE of these values has been calculated. For *E*_A_ a single pixel was measured but it was measured twice, once while increasing the heat from 300 to 340 K and once while reducing the temperature from 340 to 300 K. PCE measurement statistics are based on 16 devices for each of the PSCs.

## Conflict of Interest

The authors declare no conflict of interest.

## Supporting information



Supporting Information

Supporting Information

## Data Availability

The data that support the findings of this study are available from the corresponding author upon reasonable request.
