# Supplementary material for: Mechanical and Ionic Characterization for Organic Semiconductor‐Incorporated Perovskites for Stable 2D/3D Heterostructure Perovskite Solar Cells
Source: Small. 2024 Oct 7;20(51):2406928. doi: 10.1002/smll.202406928 (PMC11656689; doi:10.1002/smll.202406928)
Supplement: Supplementary file 1 — Supporting Information [file SMLL-20-2406928-s002.docx]

Mechanical and Ionic Characterization for Organic Semiconductor-Incorporated Perovskites for Stable 2D/3D Heterostructure Perovskite Solar Cells

*Jiaonan Sun^1†^, Saivineeth Penukula^2†^, Muzhi Li^2^, Mona Rasa Hosseinzade^1^, Yuanhao Tang^1^, Letian Dou^1,3^*, Nicholas Rolston^2^**

**Affiliations**

^1^Davidson School of Chemical Engineering, Purdue University; West Lafayette, IN 47907, USA

^2^School of Electrical, Computer and Energy Engineering, Arizona State University, Tempe, AZ 85281, USA

^3^Birck Nanotechnology Center, Purdue University; West Lafayette, IN 47907, USA

﻿^†^These authors contributed equally to this work.

*Corresponding authors. Email: [nicholas.rolston@asu.edu](mailto:nicholas.rolston@asu.edu); [dou10@purdue.edu](mailto:dou10@purdue.edu)

**Materials:**

Lead(II) iodide (99.99% trace metals basis) with a purity of 98.0% or higher and 4-isopropyl-4'-methyldiphenyliodonium tetrakis(pentafluorophenyl)borate (TPFB) were obtained from TCI America. Tin(IV) oxide (15% colloidal solution) was purchased from Alfa Aesar. Poly(triarylamine) (PTAA) with a molecular weight of 20-40k g/mol was purchased from 1-Material. Gold (Au) with a purity of 99.999% was acquired from Kurt J. Lesker. Potassium hydroxide, cesium iodide (99.999% trace metals basis), anhydrous solvents including chlorobenzene, isopropanol, dimethylformamide, dimethyl sulfoxide were from Sigma Aldrich and used directly without further purification. Formamidinium iodide, methylammonium iodide, methylammonium chloride, and n-butylammonium iodide were purchased from GreatCell Solar and used directly without further purification. 4TmI and Br4TmI were synthesized and purified based on our previous work.^[1,2]^

**Supplementary figures**


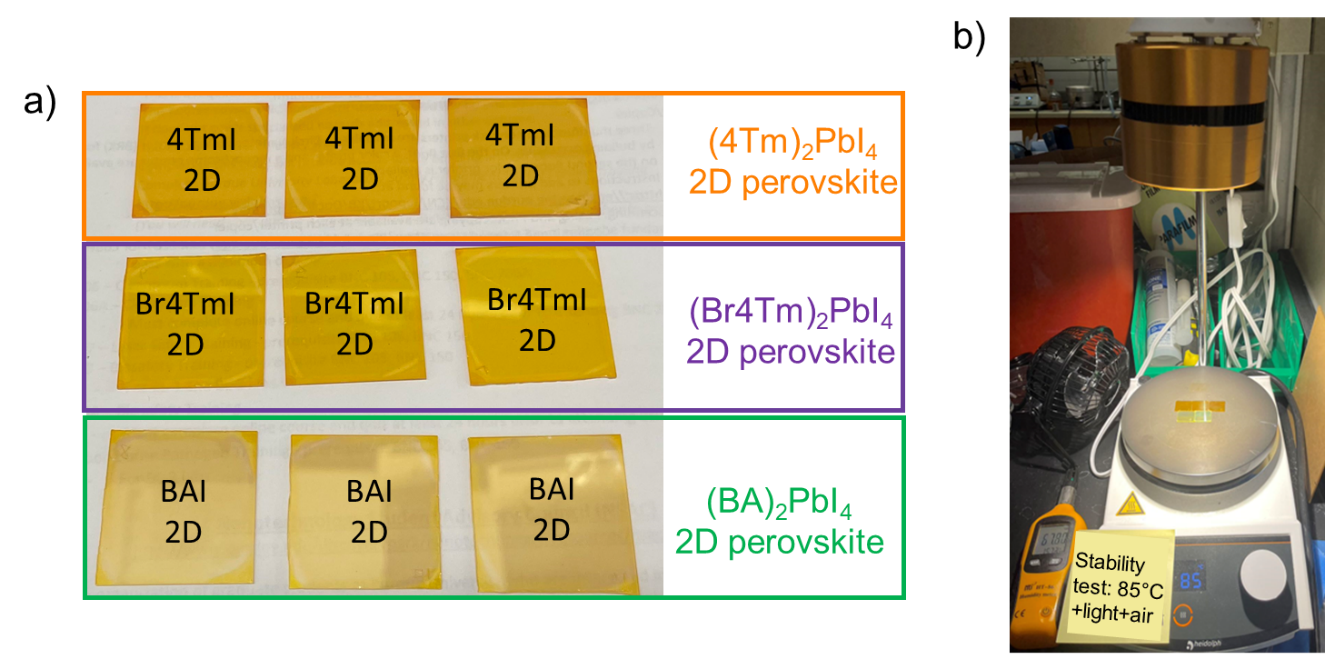


Figure S1. (a) Photograph of spin-coated 2D perovskite films. (b) Photograph experimental set-up for 2D perovskite stability tracking under 85°C heating + light (1 sun) + air (Relative Humidity (RH) %=68%).


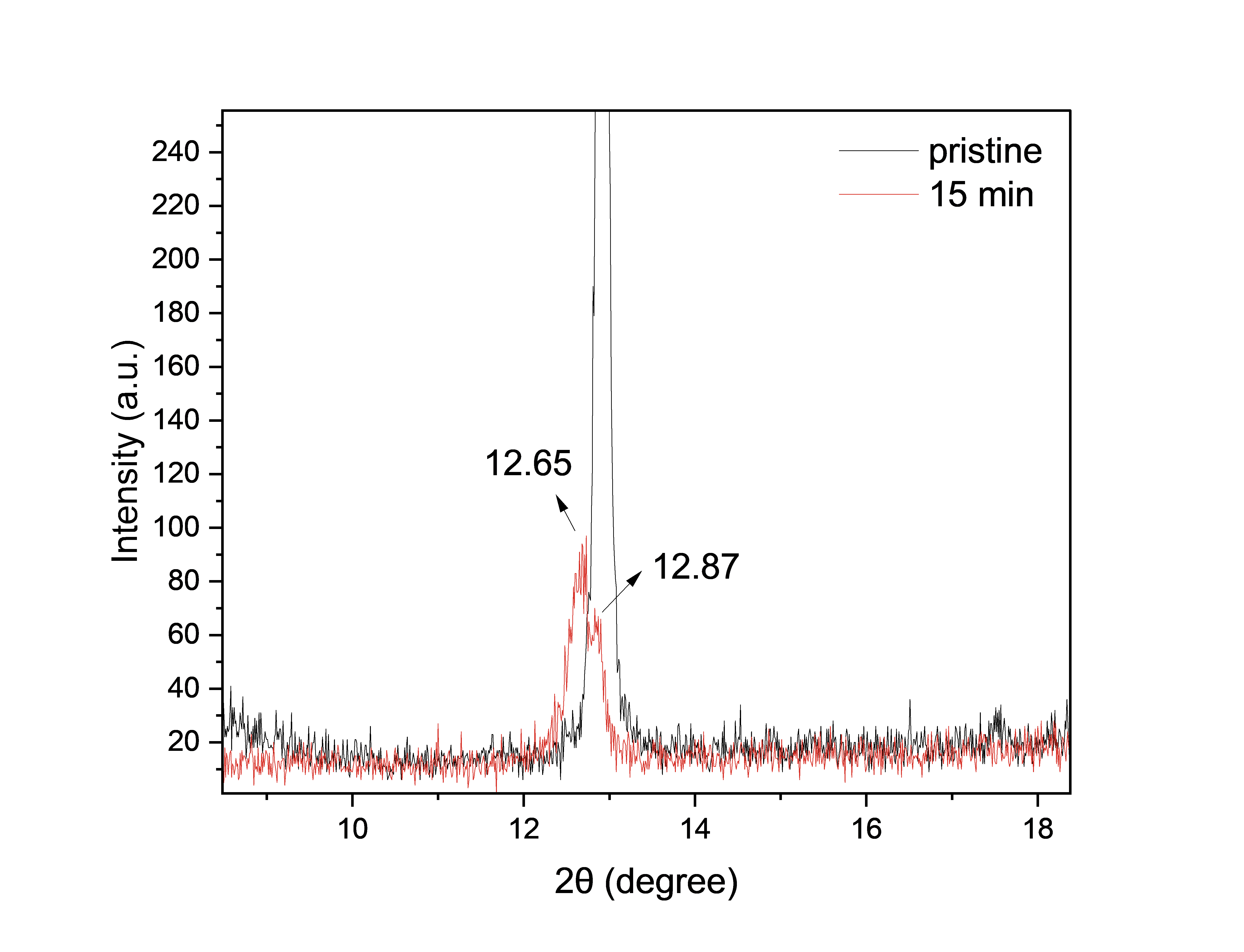
Figure S2. The enlarged XRD view of (BA)_2_PbI_4_ 2D perovskite before and after 85°C heating + light (1 sun) in air for 15 min. New peak at 12.65° is attributed to PbI_2_. This is only observed in (BA)_2_PbI_4_ 2D perovskite films. (4Tm)_2_PbI_4_ and (Br4Tm)_2_PbI_4_ 2D perovskite films did not show any PbI_2_ signal because they were still stable after the stress tests.


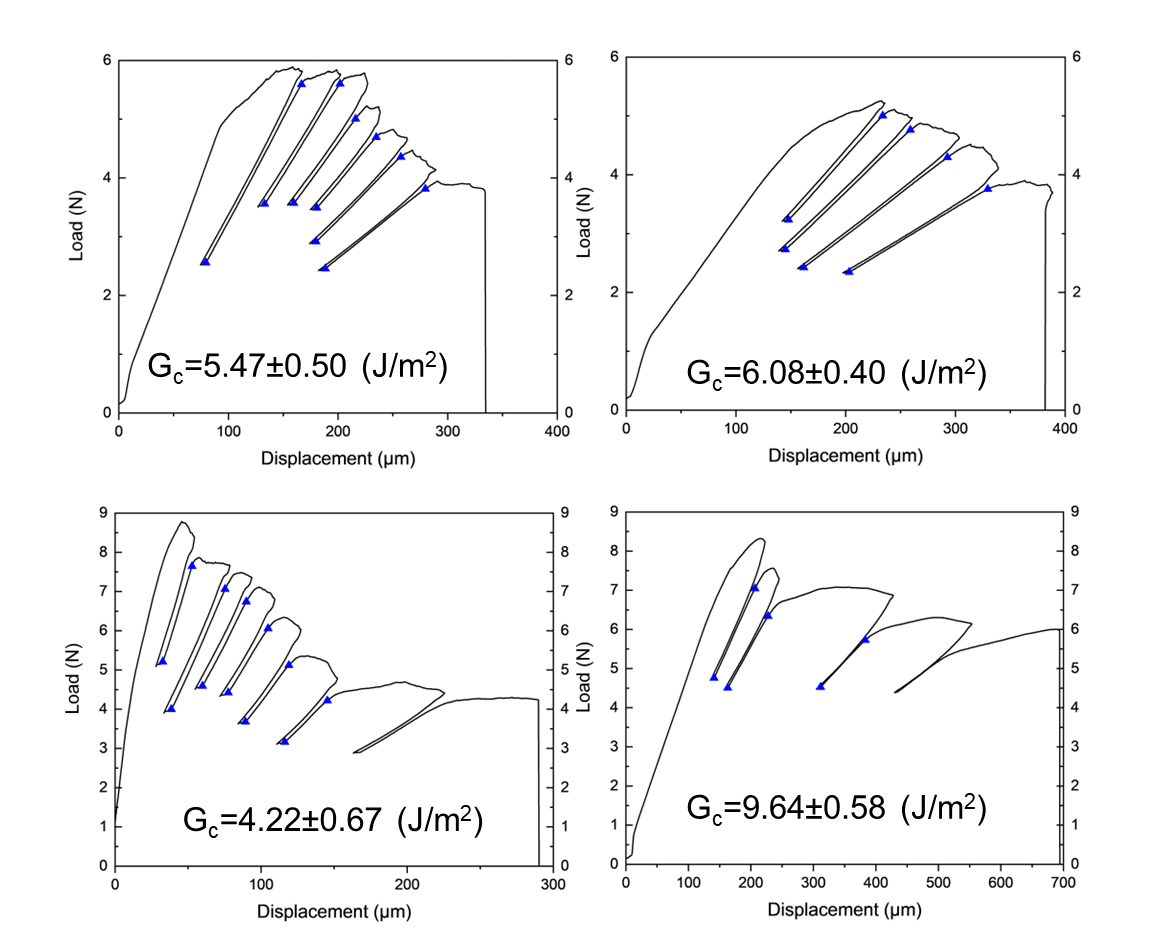


Figure S3. Loading-unloading curves of *Gc* test for 4TmI 2D thin films specimens, 4 repeated experiments


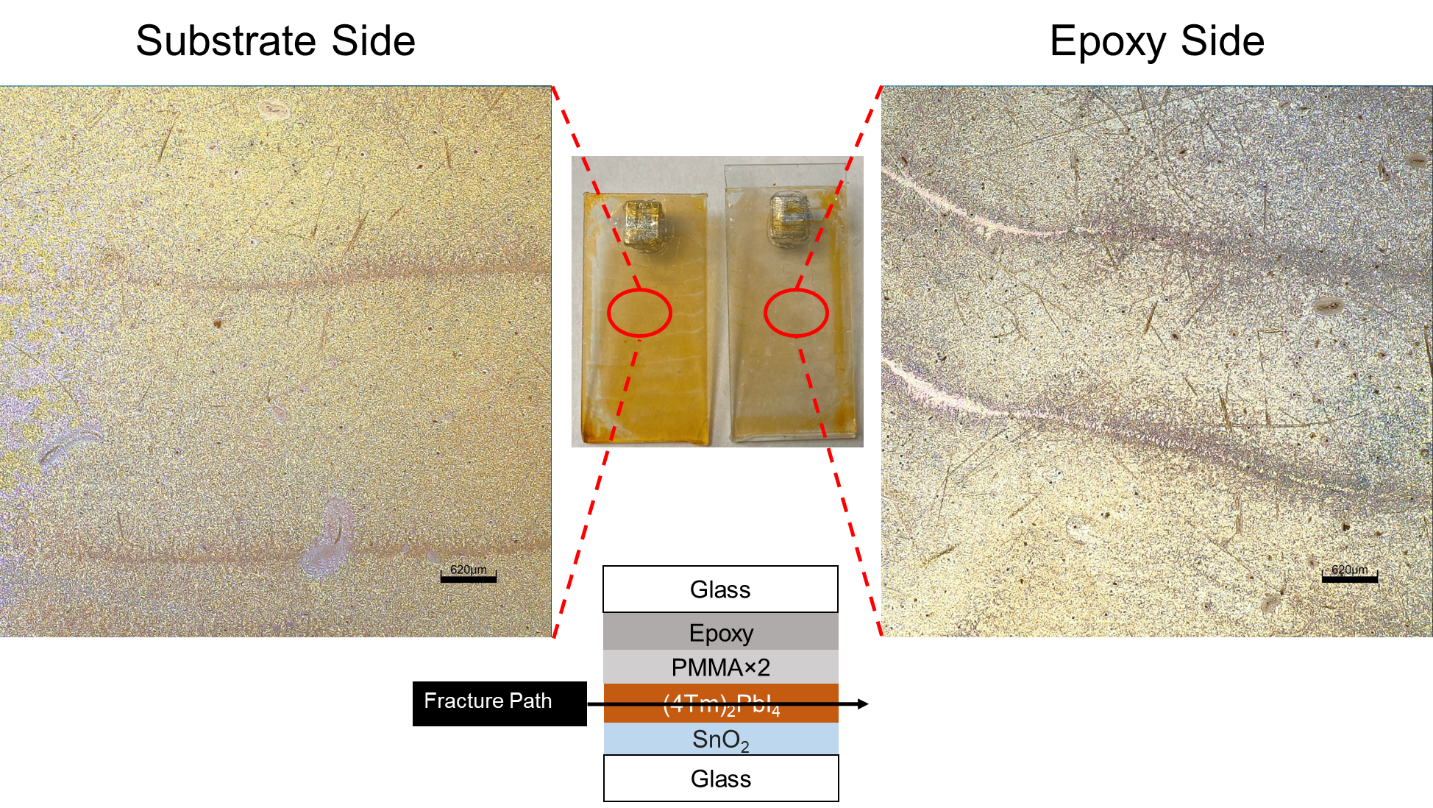


Figure S4. Representative 4Tm 2D MHP thin film specimen after *Gc* measurement, with microscope images on both sides.


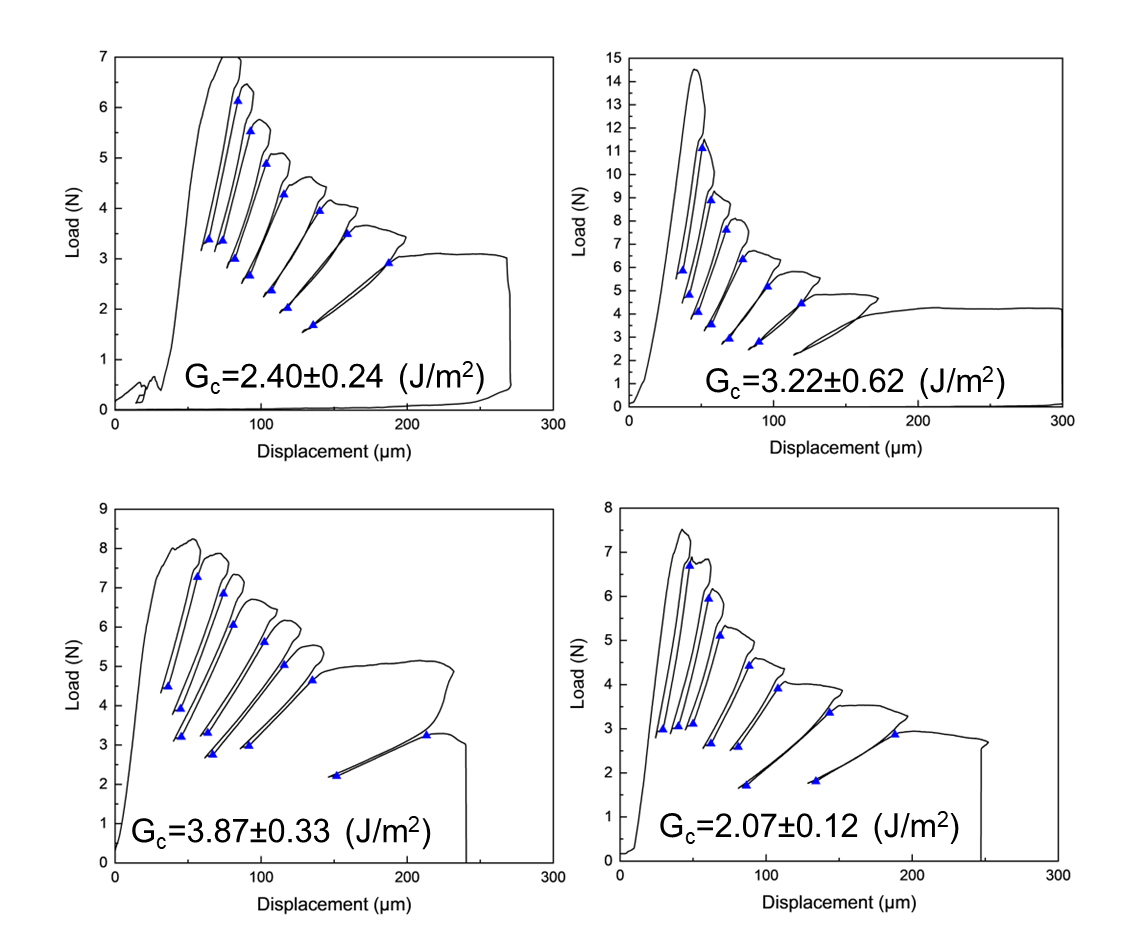


Figure S5. Loading-unloading curves of *Gc* test for Br4TmI 2D thin films specimens, 4 repeated experiments


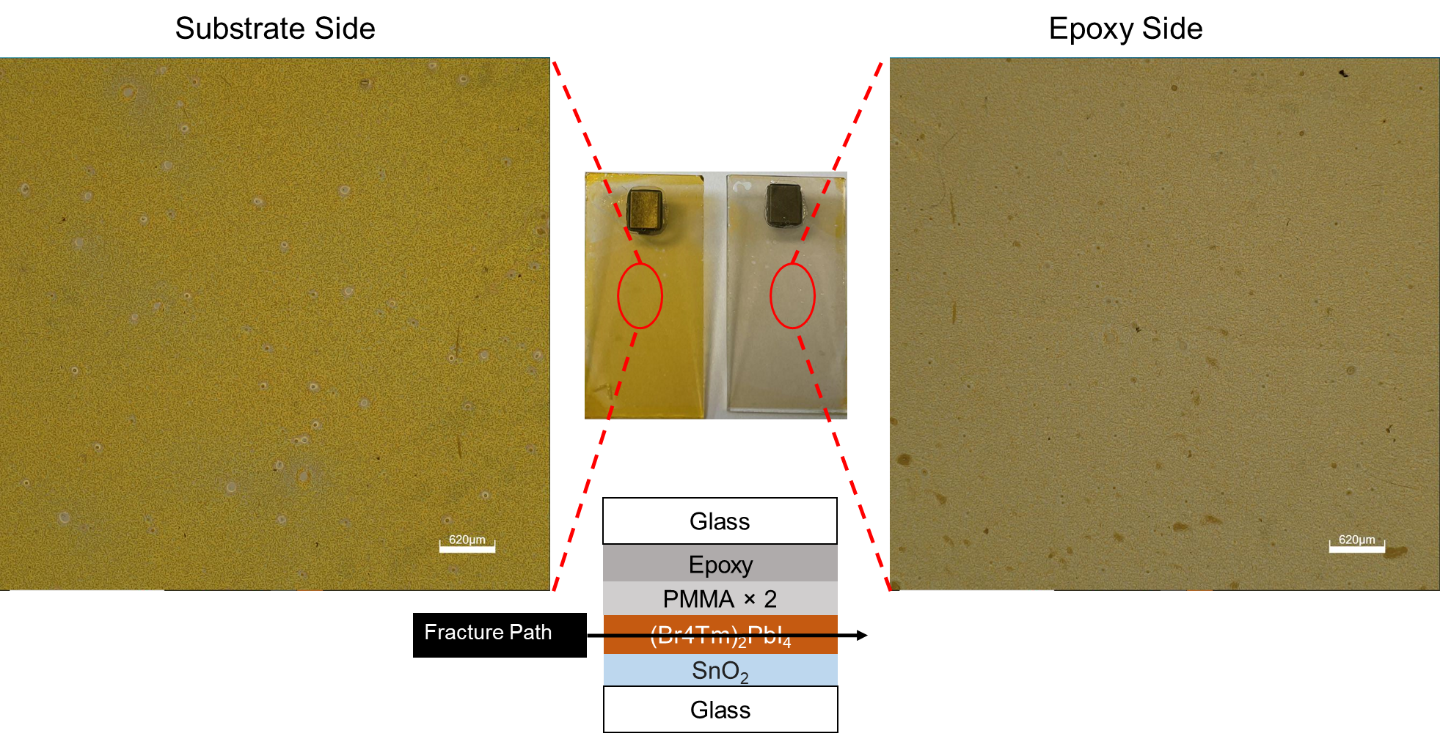


Figure S6. Representative Br4TmI 2D MHP thin film specimen after *Gc* measurement, with microscope images on both sides.


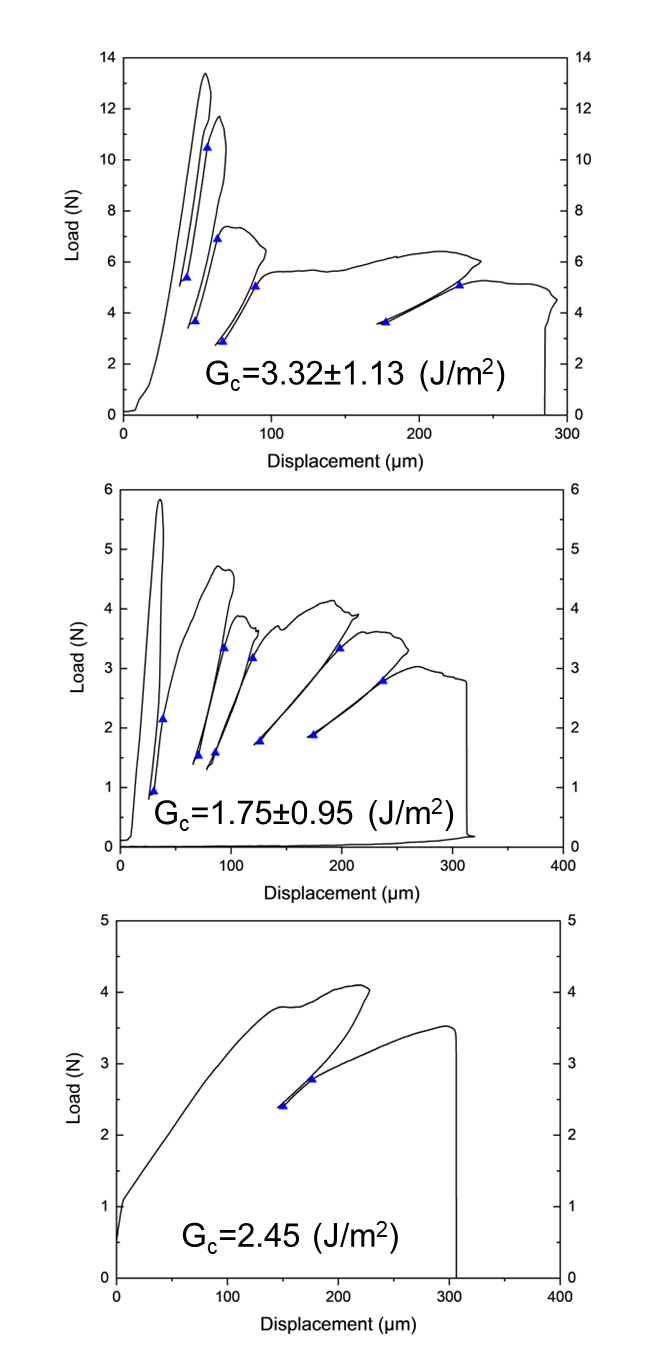


Figure S7. Loading-unloading curves of *Gc* test for BAI 2D thin films specimens, 3 repeated experiments.


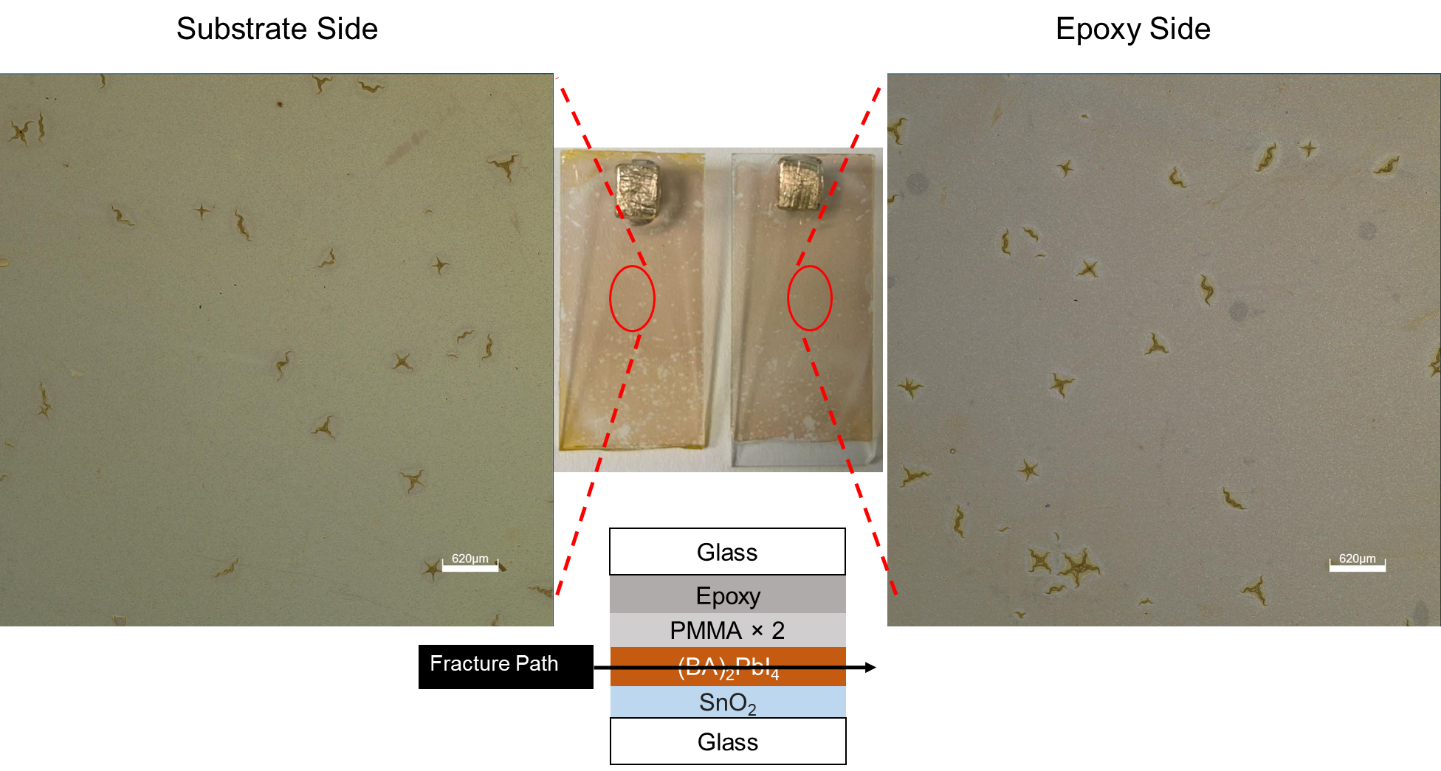


Figure S8. Representative BAI 2D MHP thin film specimen after *Gc* measurement, with microscope images on both sides.


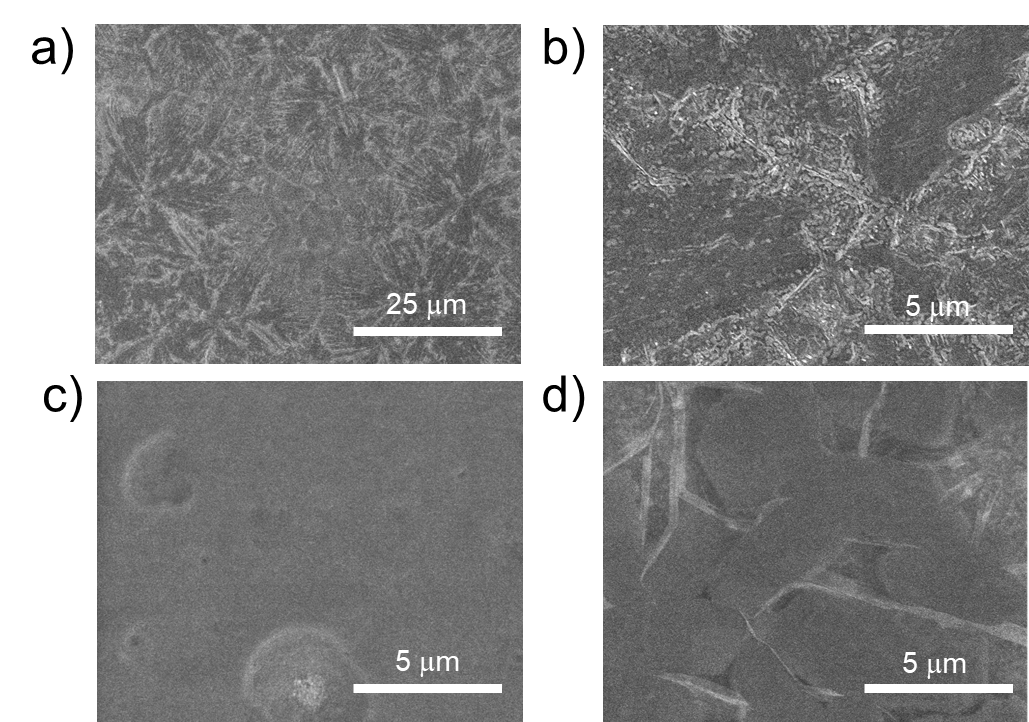


Figure S9. SEM images of 2D (a-b) (BA)_2_PbI_4_, (c) (Br4Tm)_2_PbI_4_, and (d) (4Tm)_2_PbI_4_ thin films coated on ITO/SnO_2_.


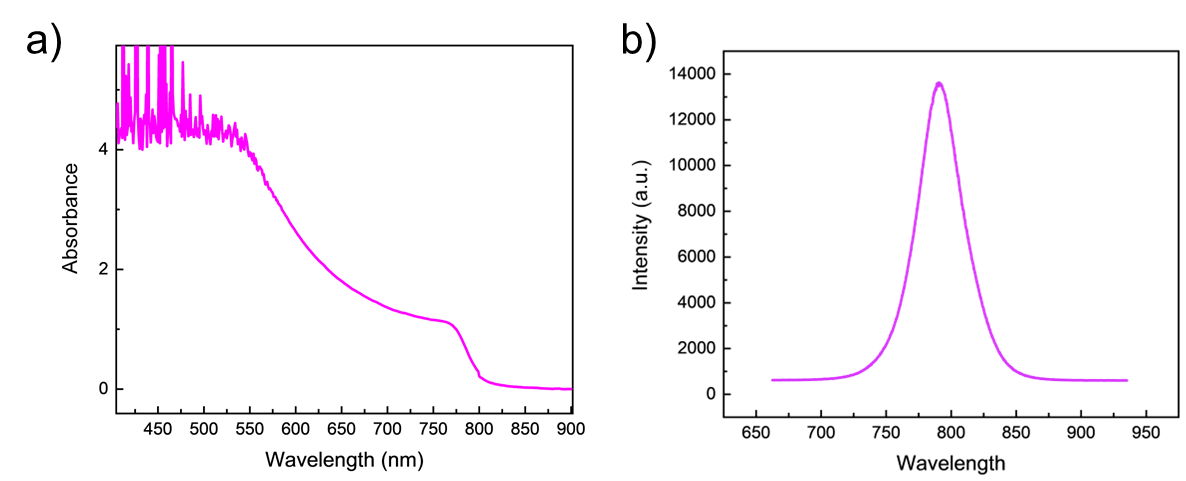


Figure S10. a) UV-vis and b) Photoluminescence of the perovskite materials in devices.


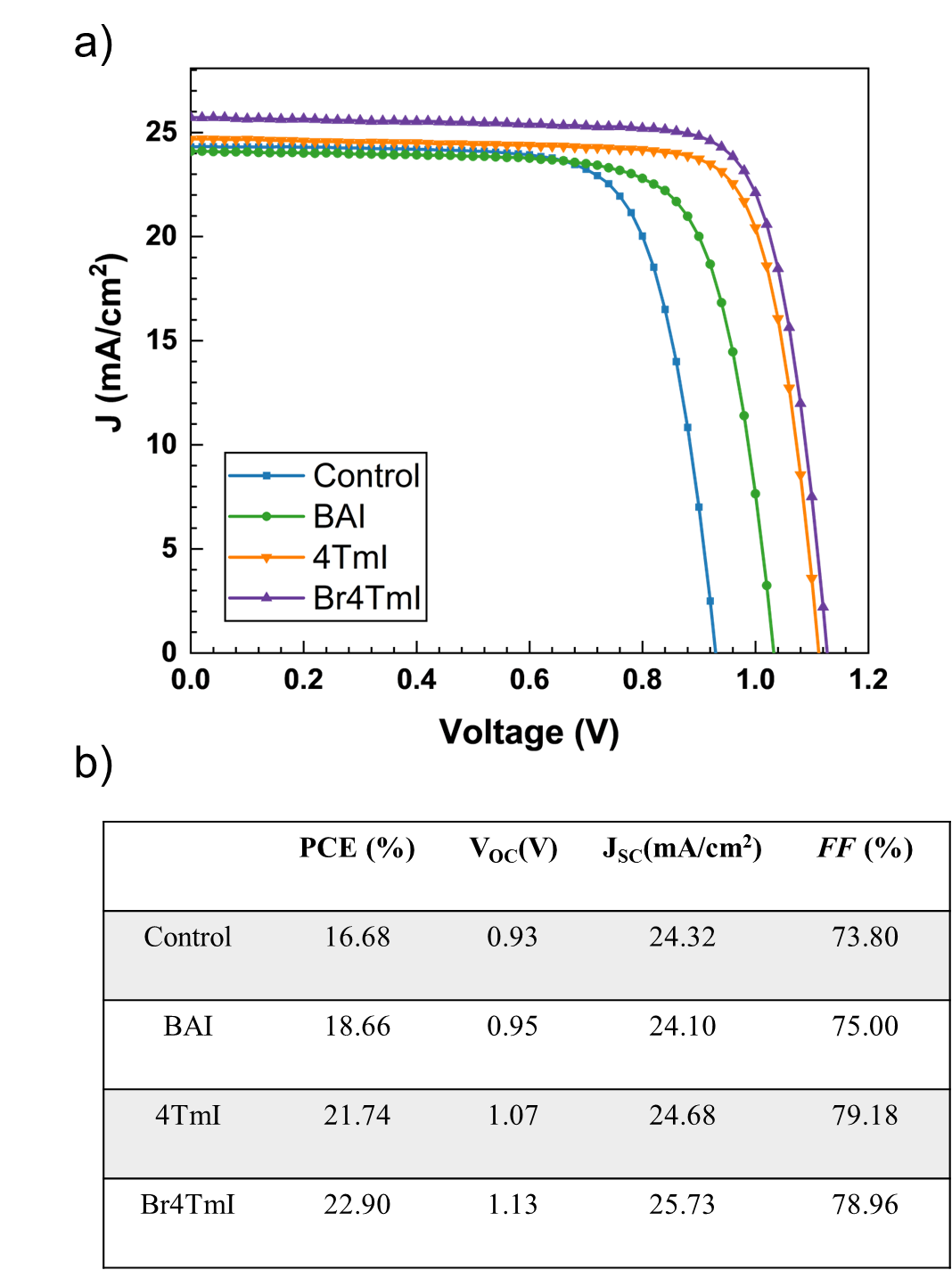


Figure S11. a) Representative J-V curves and b) device parameters of control, BAI, 4TmI, Br4TmI


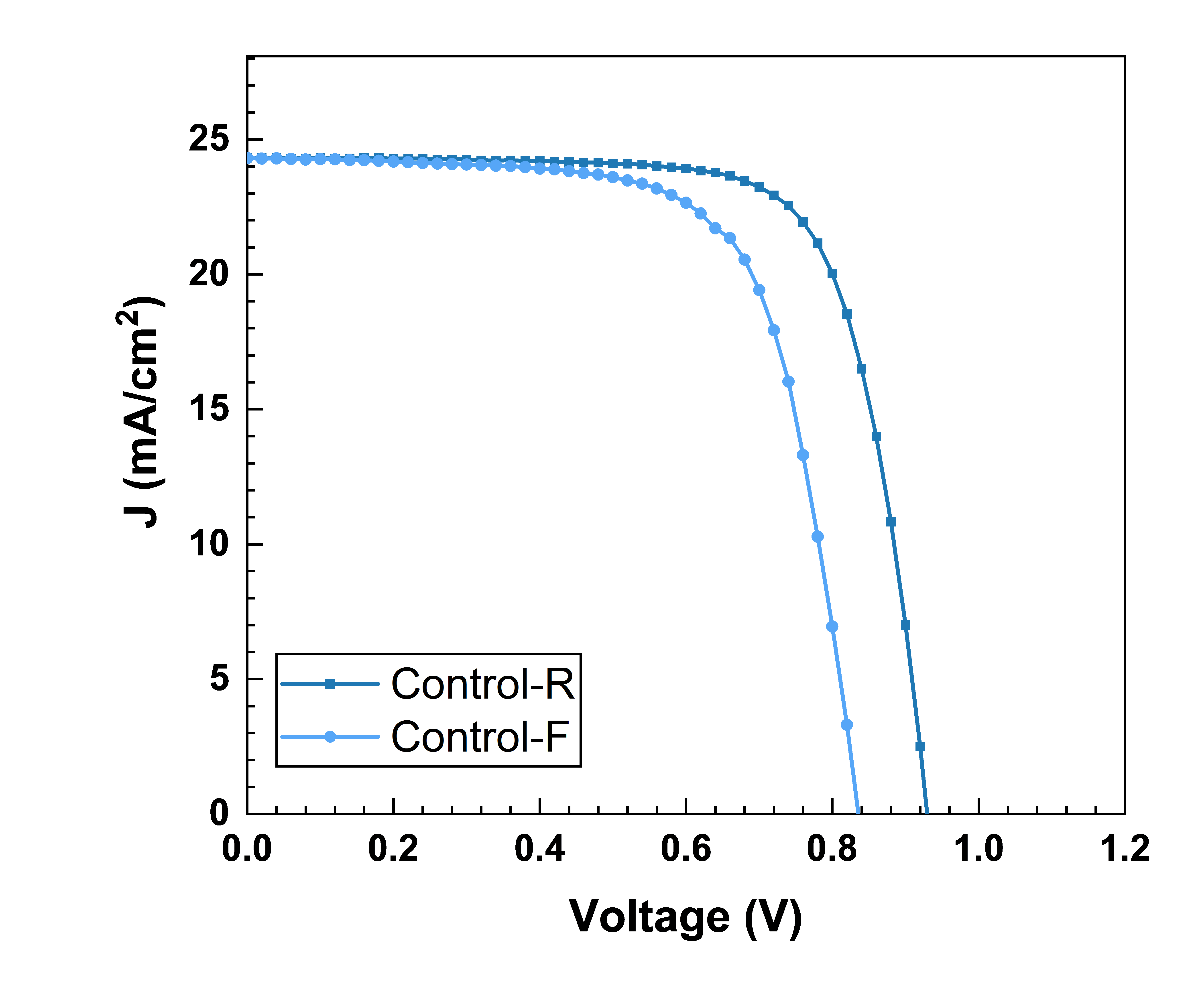


Figure S12. Representative J-V curves of control devices, with both reverse and forward scan.


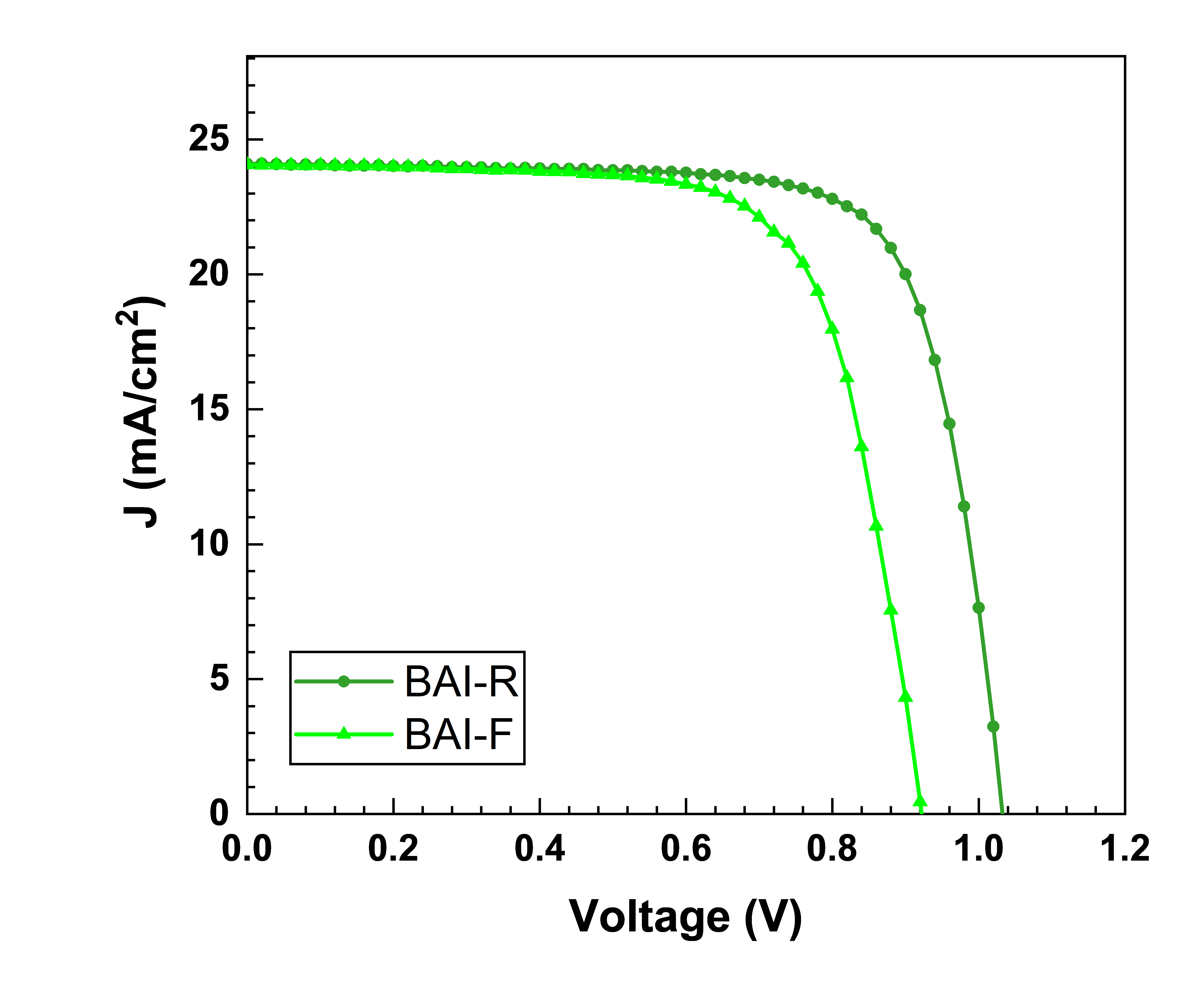


Figure S13. Representative J-V curves of BAI devices, with both reverse and forward scan.


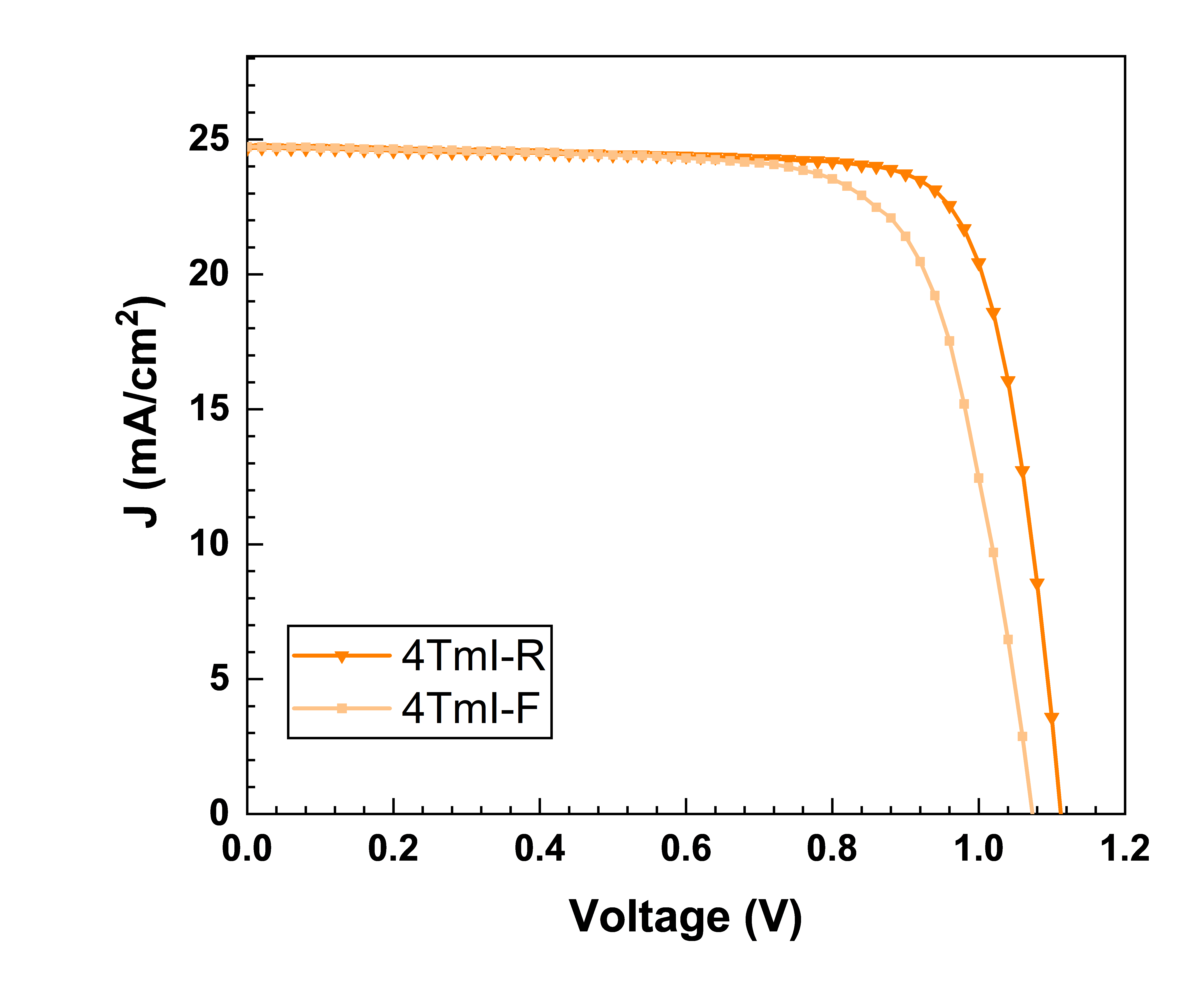


Figure S14. Representative J-V curves of 4TmI devices, with both reverse and forward scan.


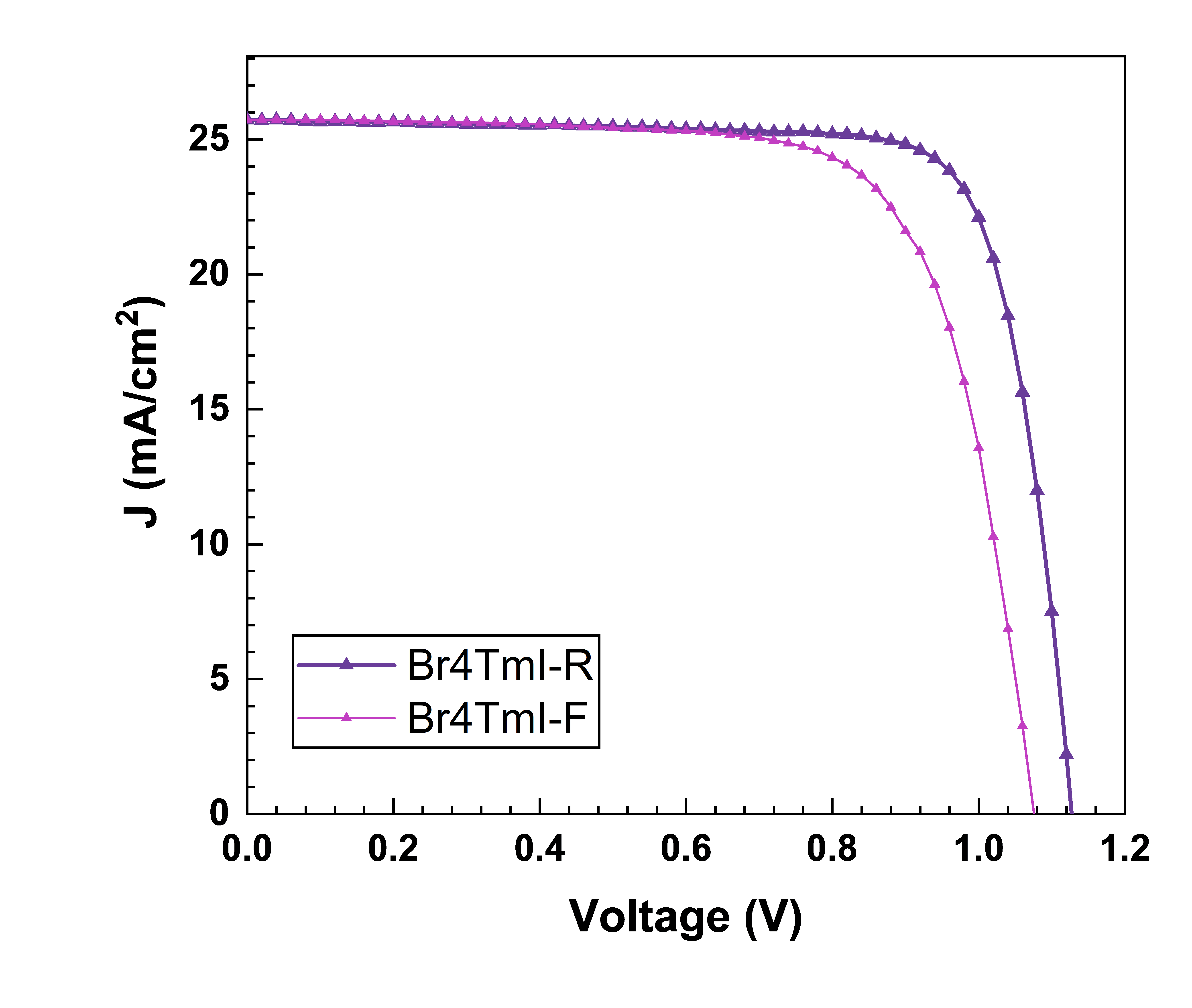


Figure S15. Representative J-V curves of Br4TmI devices, with both reverse and forward scan.


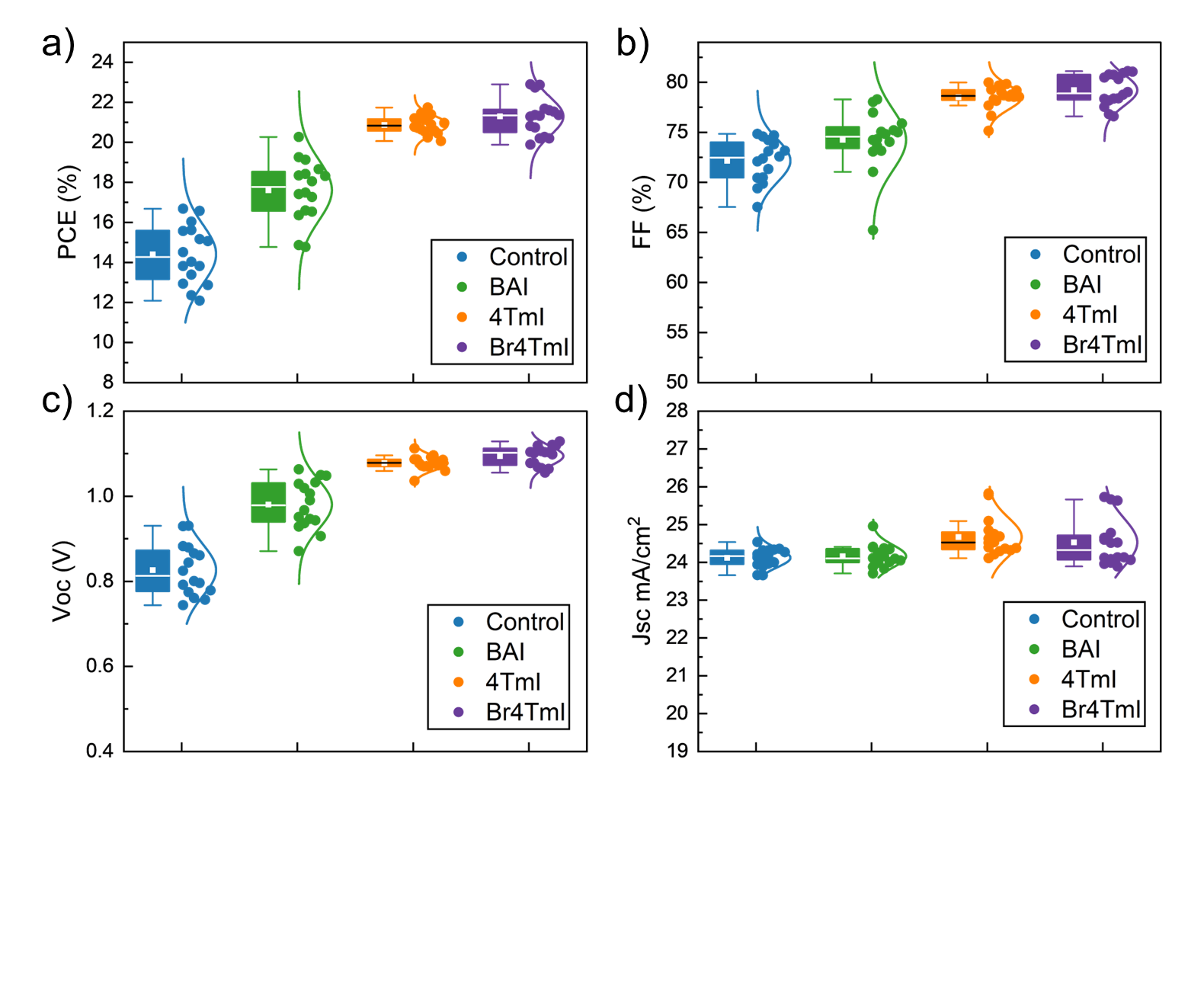


Figure S16. Device statistics of a) PCE b) *FF* c) V_OC_ d) J_SC_. Statistics are based on 16 devices.


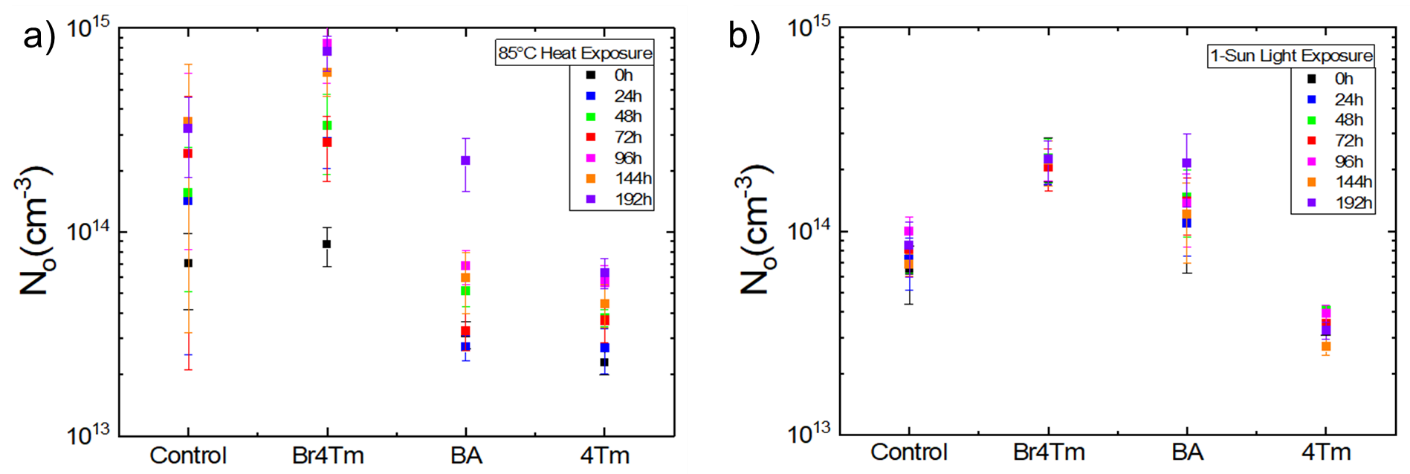


Figure S17. N_o_ VS PSC with aging for 192h when subjected to a) 85°C heat showing the least change in N_o_ for PSC with 4Tml 2D interlayer based on the clustering of N_o_, and b) 1-Sun light showing the least change in N_o_ for PSC with Br4Tml 2D interlayer and second least change in N_o_ for PSC with 4Tml 2D interlayer based on the clustering of N_o_


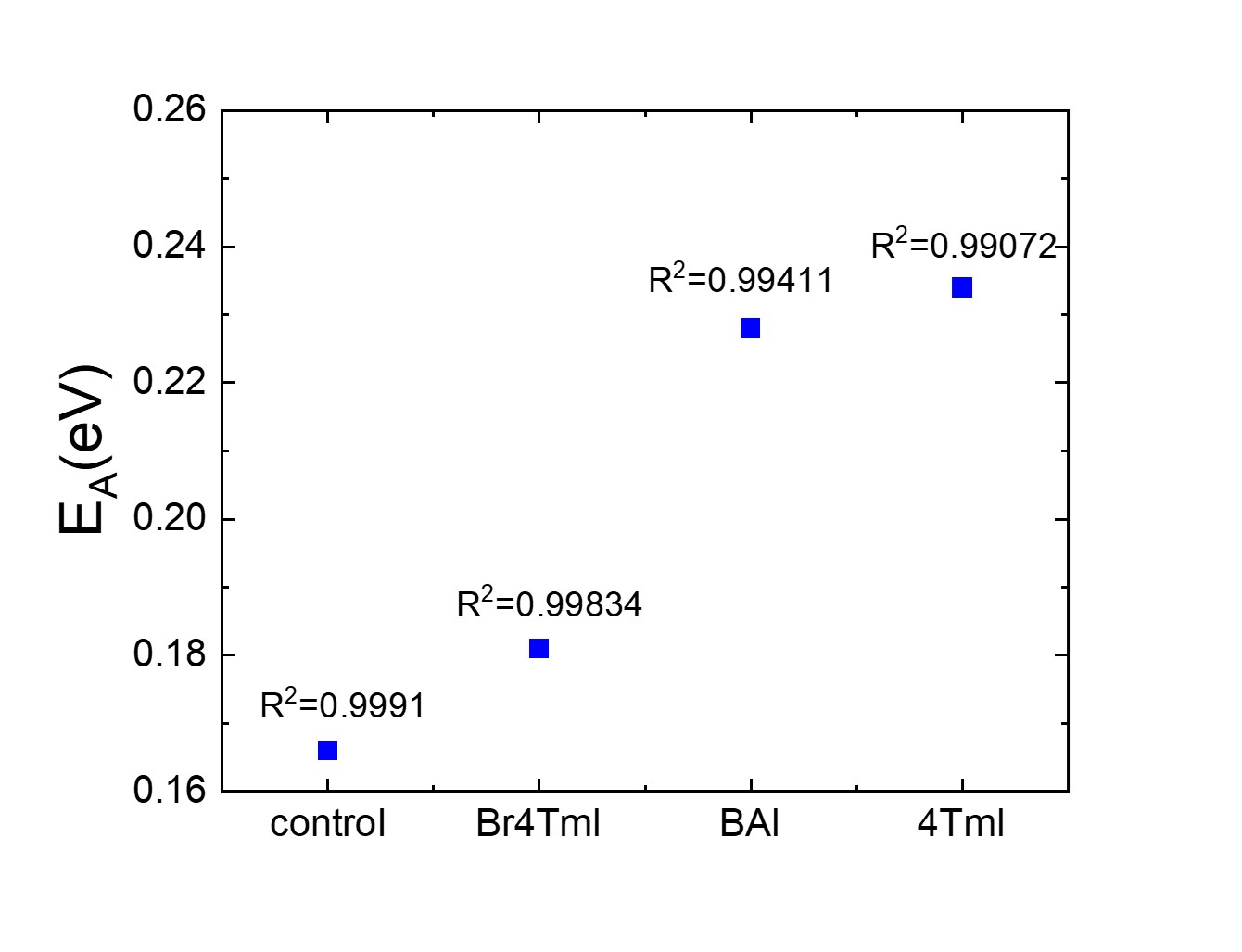


Figure S18. Activation energy of the PSCs with R-square values


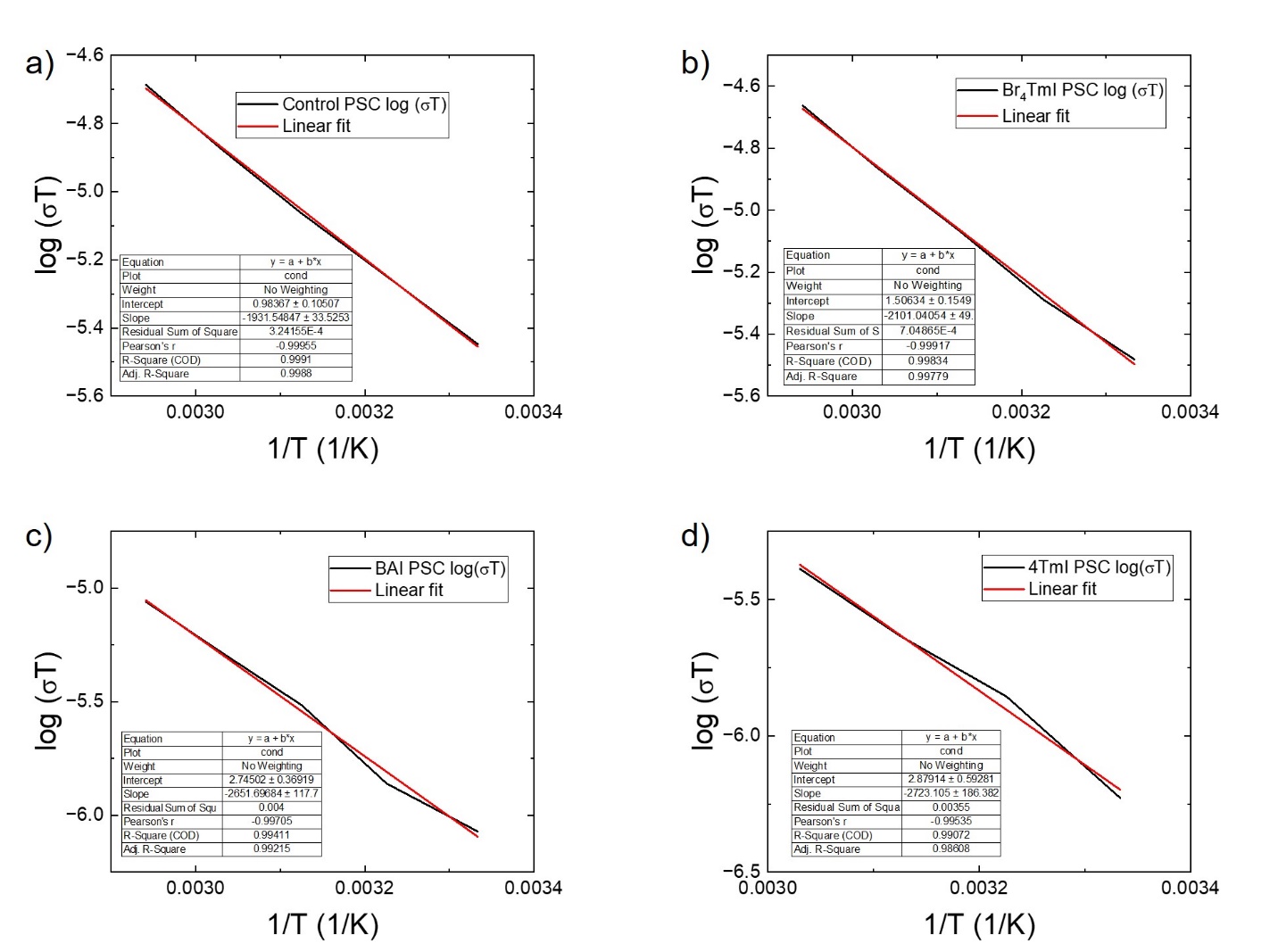


Figure S19. Activation energy fits of a) Control PSC, b) PSC with Br4TmI 2D interlayer, c) PSC with BAI 2D interlayer, and d) PSC with 4TmI 2D interlayer


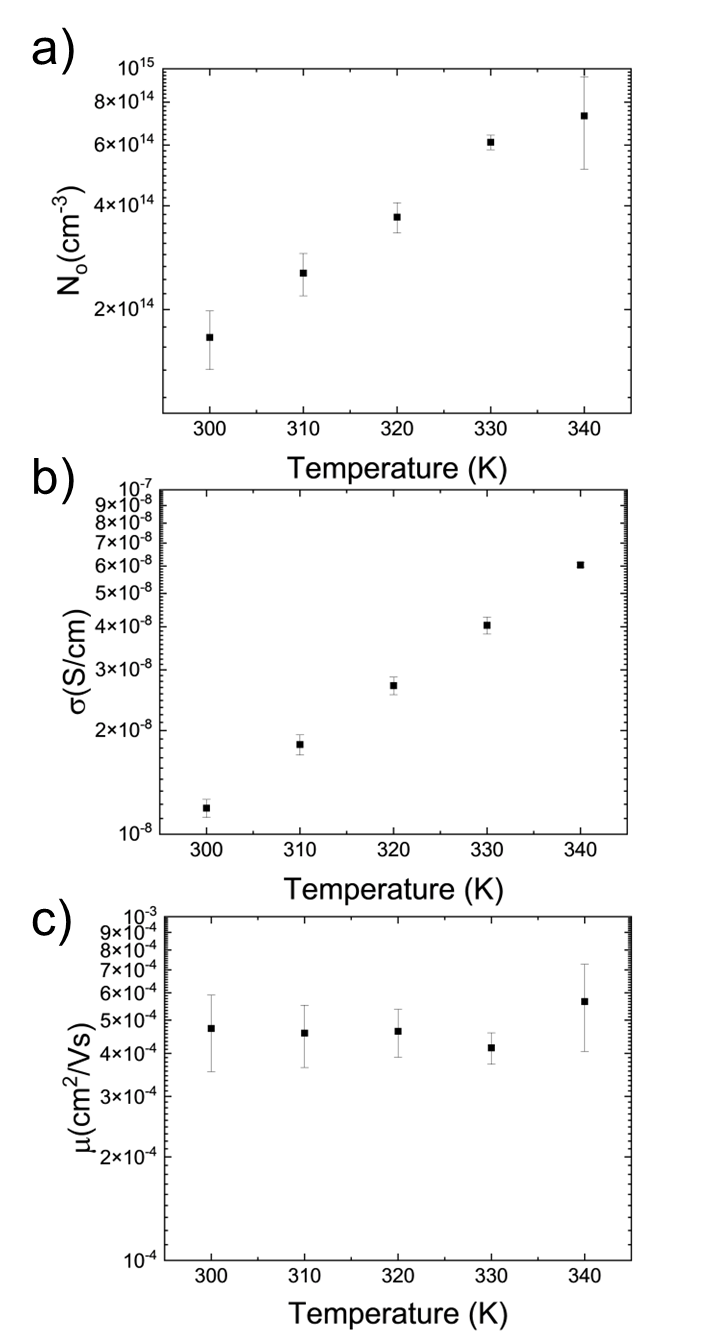


Figure S20. *In-situ*Ionic measurements vs Temperature of control PSC a) mobile ion concentration b) ionic conductivity c) ionic mobility


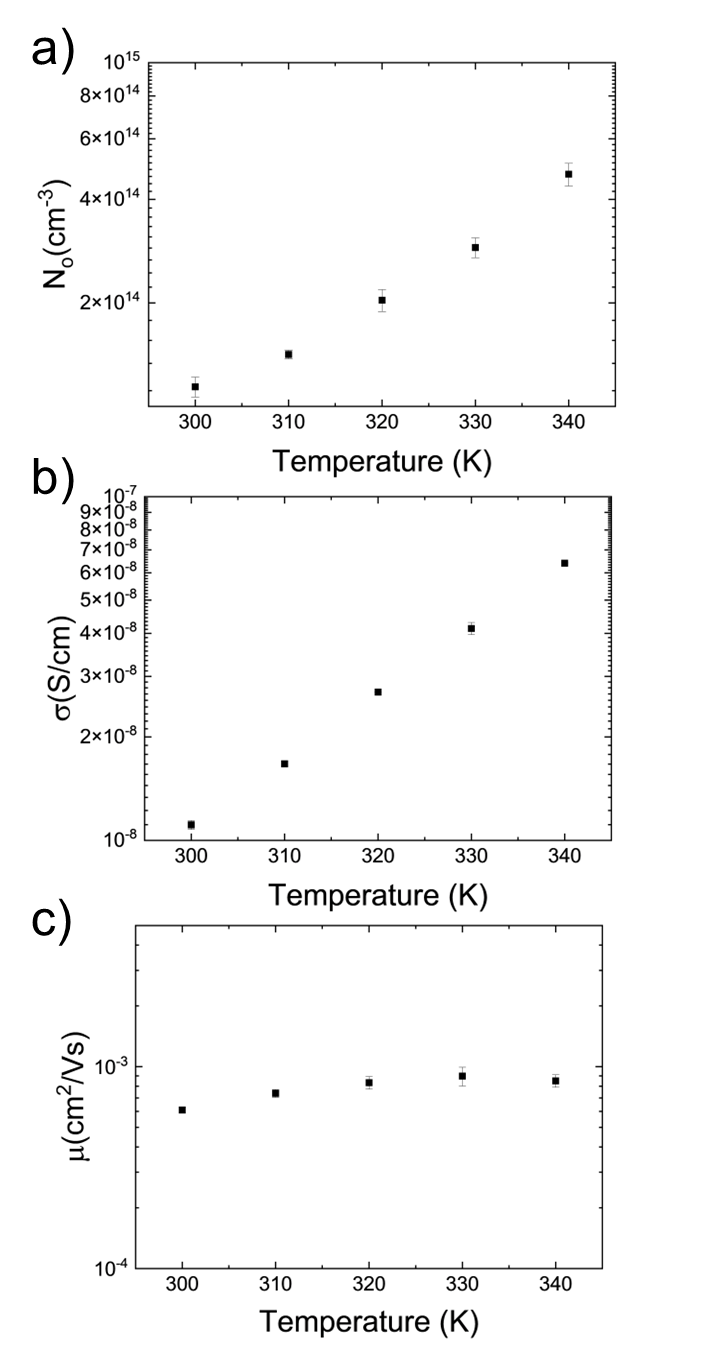


Figure S21. *In-situ*Ionic measurements vs Temperature of PSC with Br4Tml 2D interlayer a) mobile ion concentration b) ionic conductivity c) ionic mobility


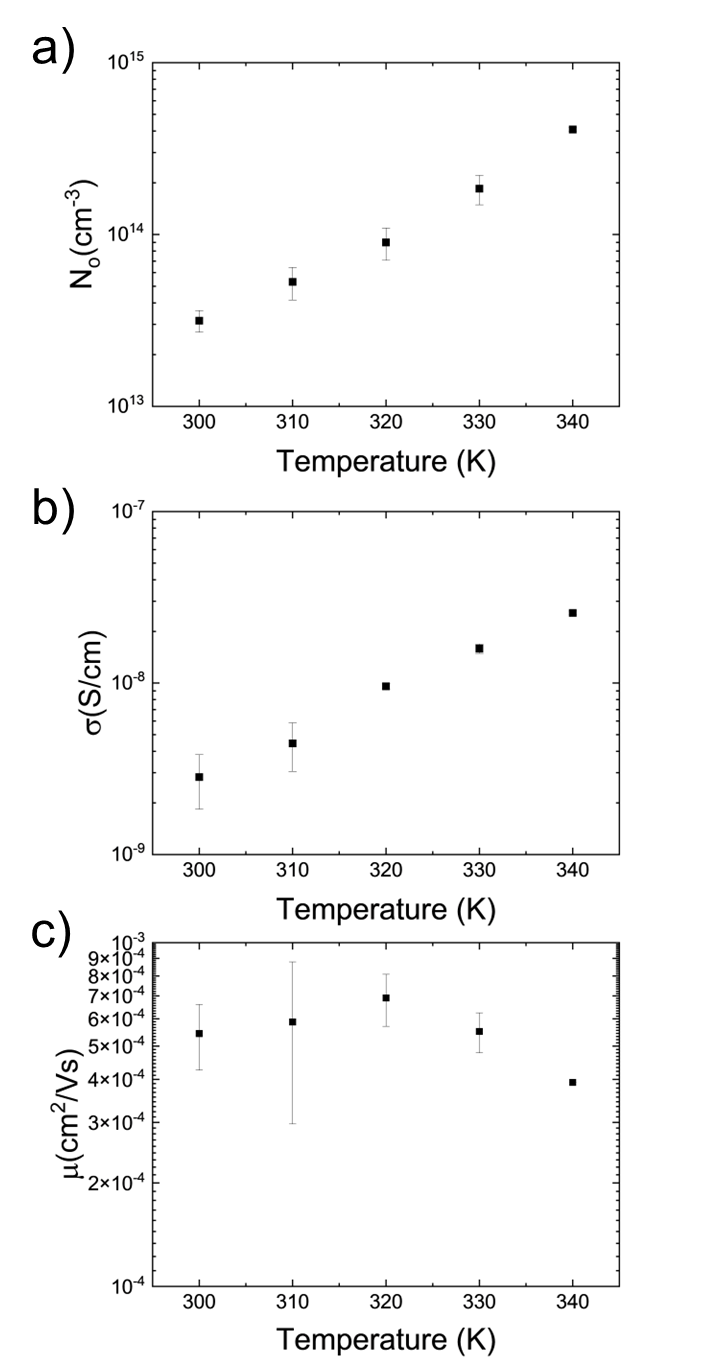


Figure S22. *In-situ*Ionic measurements vs Temperature of PSC with BAI 2D interlayer a) mobile ion concentration b) ionic conductivity c) ionic mobility


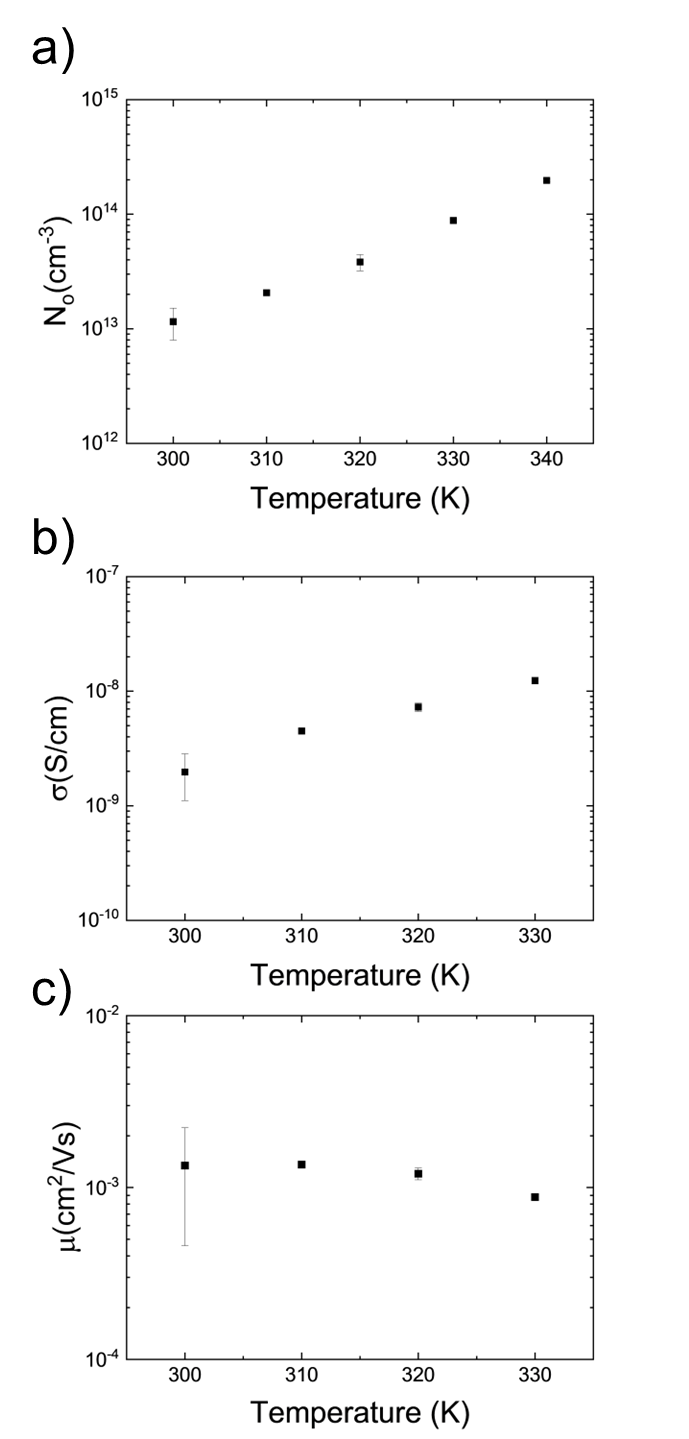


Figure S23. *In-situ*Ionic measurements vs Temperature of PSC with 4TmI 2D interlayer a) mobile ion concentration b) ionic conductivity c) ionic mobility

Table S1. Stability summary for 2D MHPs and 2D/3D heterostructure PSCs, ranking 1 to 4: best to least.


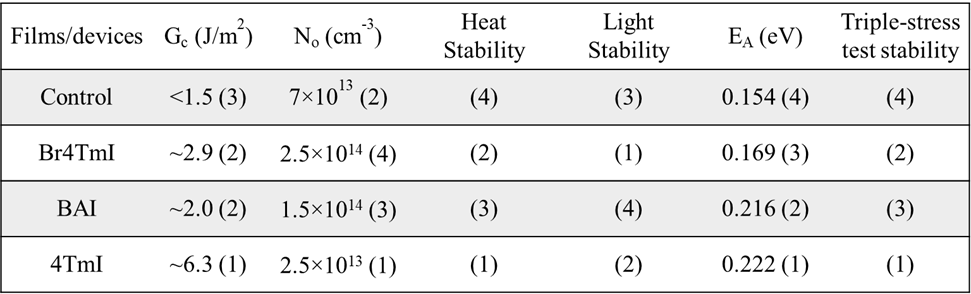


**References**

[1] K. Ma, J. Sun, R. H. Atapattu, W. B. Larson, H. Yang, D. ; Sun, K. Chen, K. Wang, Y. Lee, Y. Tang, A. Bhoopalam, L. Huang, R. K. Graham, J. Mei, L. Dou, *Sci Adv* **2023**, *9*, eadg0032.

[2] Y. Gao, E. Shi, S. Deng, S. B. Shiring, J. M. Snaider, C. Liang, B. Yuan, R. Song, S. M. Janke, A. Liebman-Peláez, P. Yoo, M. Zeller, B. W. Boudouris, P. Liao, C. Zhu, V. Blum, Y. Yu, B. M. Savoie, L. Huang, L. Dou, *Nat Chem* **2019**, *11*, 1151.
